# Double reflex testing for anti-HDV antibody following an HBsAg-positive test result and HDV RNA in those anti-HDV positive: a global systematic review and meta-analysis

**DOI:** 10.1016/j.eclinm.2025.103708

**Published:** 2026-01-05

**Authors:** Feifei Li, Yusha Tao, Chengxin Fan, Yifan Dai, Jamie L. Conklin, Joseph D. Tucker, Roger Chou, Philippa Easterbrook, Weiming Tang

**Affiliations:** a Southern Medical University Institute for Global Health, Guangzhou, China; b Dermatology Hospital of Southern Medical University, Guangzhou, China; c University of North Carolina Project-China, Guangzhou, China; d School of Public Health, Nanjing Medical University, Nanjing, China; e MRC Centre for Global Infectious Disease Analysis, School of Public Health, Imperial College London, London, UK; f Health Sciences Library, University of North Carolina at Chapel Hill, Chapel Hill, NC, USA; g Institute of Global Health and Infectious Diseases, University of North Carolina at Chapel Hill, Chapel Hill, USA; h Clinical Research Department, Faculty of Infectious and Tropical Diseases, London School of Hygiene and Tropical Medicine, London, UK; i Oregon Health & Science University, Oregon, USA; j Department of Global HIV, Hepatitis and Sexually Transmitted Infections, WHO, Geneva, Switzerland; k Division of Digestive Diseases, Department of Metabolism, Digestion and Reproduction, Imperial College London, London, UK

**Keywords:** Hepatitis delta, Reflex testing, Systematic review

## Abstract

**Background:**

Chronic hepatitis D (CHD) coinfection leads to accelerated progression to liver cirrhosis, hepatocellular carcinoma, and increased mortality. Currently, only a small proportion of individuals who are HBsAg positive are tested for anti-HDV antibodies, and among those who test positive, few receive confirmatory HDV RNA testing. Reflex testing, whereby laboratory-based anti-HDV testing is automatically triggered with a positive HBsAg test result in the lab, and also HDV RNA testing in those with a positive anti-HDV result, is one approach to promote the uptake and earlier diagnosis of HDV coinfection. We undertook a systematic review and meta-analysis to evaluate the effectiveness of a laboratory-based reflex testing strategy for both anti-HDV and HDV RNA on uptake of testing and linkage to care and turnaround times across the care cascade.

**Methods:**

We searched five databases (PubMed, Scopus, Embase, Cochrane, and Global Health (EBSCOhost)) and conference abstracts for relevant studies from database inception through June 2025, with or without a non-reflex comparator arm, and that had data on at least one step in the care cascade (uptake of HDV antibody and HDV RNA testing, linkage to care, and treatment initiation). Summary estimates were calculated using random-effects meta-analyses with 95% confidence intervals (CIs). The strength of evidence was assessed using the GRADE framework. The protocol was registered with PROSPERO (CRD42023397577).

**Findings:**

Of 3160 studies identified in the search, 28 were eligible for inclusion, of which 9 also had a non-reflex comparison arm. The majority (82.1%) were conducted in high-income countries. Among the 28 studies that used a reflex testing, 97.1% (95% CI: 93.9–98.7) of HBsAg-positive individuals had anti-HDV testing, and 7.2% (95% CI: 5.8–8.9) were anti-HDV positive. Of these, 95.3% (95% CI: 90.1–97.8) had HDV RNA testing, and 43.2% (95% CI: 33.2–53.8) were RNA positive. In the nine studies with a non-reflex comparison arm, reflex testing significantly increased anti-HDV testing uptake (98.0% versus 39.7%; RR 2.55, 95% CI: 1.62–4.05). And in the six studies with a comparator arm that also examined HDV RNA testing, uptake was higher with reflex testing but this was not statistically significant (94.7% versus 79.9%; RR 1.02, 95% CI: 0.86–1.20). Nine studies reported turnaround times for reflex testing from HBsAg testing to anti-HDV reflex testing, and all were completed within the same day. Four studies also reported a same-day turnaround for HDV RNA testing. Linkage to care was reported in only three of the reflex testing studies, and all 6 HDV RNA-positive individuals were referred for care.

**Interpretation:**

Laboratory-based reflex testing significantly increased the uptake of anti-HDV antibody and HDV RNA testing across diverse populations and settings, with same day turnaround times. The 2024 World Health Organization guidelines for hepatitis B care and treatment now recommend reflex HDV testing as a scalable approach to improve HDV testing uptake, and linkage to care for surveillance for liver cancer, and access to new treatments for Delta coinfection.

**Funding:**

The Guangzhou Science and Technology Project; The National Key R& D Program; 10.13039/100000002National Institutes of Health; The University of North Carolina Center for AIDS Research; 10.13039/100004423World Health Organization.

**Research in context**

**Evidence before this study**

We searched PubMed, Scopus, Embase, Cochrane, and Global Health (EBSCOhost) from database inception to June 2025, without language restrictions, and also reviewed abstracts from major international conferences. We used combinations of search terms that included “hepatitis D virus”, “HDV”, “reflex testing”, “HBsAg”, and “care cascade”. Studies were eligible if they reported outcomes related to anti-HDV antibody and HDV RNA testing uptake, linkage to care, or treatment initiation, with or without a non-reflex comparator arm. Previous systematic reviews have addressed aspects of hepatitis B service delivery, but none to our knowledge have comprehensively assessed reflex HDV testing strategies across the care cascade, nor provided pooled estimates of their effectiveness.

**Added value of this study**

To our knowledge, this is the first systematic review and meta-analysis to comprehensively assess reflex HDV testing globally. By synthesizing data from 28 studies across diverse populations and settings, we found that reflex testing substantially increases uptake of both anti-HDV antibody and HDV RNA testing, often with same-day turnaround times. These findings provide evidence that reflex testing is a practical and effective strategy to improve early diagnosis and linkage to care for people with HDV coinfection.

**Implications of all the available evidence**

Our findings support the inclusion of reflex HDV testing as a key strategy in global hepatitis elimination efforts. Implementation of reflex testing could substantially improve case detection, facilitate improved surveillance for liver cancer among a high-risk population of persons with HDV coinfection, and accelerate access to emerging antiviral therapies for HDV infection. Policymakers and health systems should consider integrating reflex HDV testing into routine hepatitis B management pathways to close critical gaps in the care cascade.

## Introduction

Hepatitis delta virus (HDV) is a single-stranded RNA virus that replicates in the presence of hepatitis B virus (HBV).^1^ Chronic hepatitis D (CHD) coinfection leads to accelerated progression to liver cirrhosis, hepatocellular carcinoma, and increased mortality. Hepatitis D virus (HDV) poses a significant global health burden, especially in areas where hepatitis B virus (HBV) is highly endemic.^2^ Recently updated estimates of HDV prevalence based on 25 countries reveal striking regional disparities, with the highest rates observed in the Eastern Mediterranean, Africa, and the Americas, and substantially lower levels in South-East Asia and the Western Pacific.^3^ However, globally, only a small proportion of those who are HBsAg positive are tested for HDV infection coinfection (anti-HDV antibody) and then if positive tested for HDV RNA to diagnose viraemic HDV infection.^4^ Timely identification of coinfected individuals is essential, particularly in regions with a high burden of chronic hepatitis B (CHB), to provide optimal clinical management that includes both enhanced surveillance for HCC and assessment for eligibility for existing and newer treatment options.^5^ Early detection of HDV can improve clinical outcomes through timely counselling, antiviral treatment for CHB and Delta coinfection as indicated, surveillance for HCC, and HBV vaccination of family members to prevent HBV transmission.

Diagnosis of HDV typically follows a two-step process. First, anti-HDV antibodies are tested in individuals who are positive for hepatitis B surface antigen (HBsAg). Then, those who test positive for anti-HDV undergo HDV RNA testing to confirm viremic infection.^6^ While anti-HDV positivity indicates exposure, only RNA testing confirms ongoing viral replication and potential eligibility for antiviral therapy for HDV infection. Although international guidelines from both the European Association for the Study of the Liver (2017; updated in 2023) and the Asian–Pacific Association for the Study of the Liver (2016) recommend anti-HDV testing for all individuals who test positive for HBsAg, real-world implementation at the national level remains rare due to limited clinician awareness, lack of integration of HDV testing into national screening or reimbursement frameworks, and insufficient laboratory capacity for confirmatory HDV RNA testing in many regions.^7^^,^^8^ Furthermore, when anti-HDV testing is performed, confirmatory HDV RNA testing is often delayed or not conducted at all. These gaps represent missed opportunities to initiate appropriate care and reduce transmission risk for people living with HDV.

Laboratory-based reflex testing, in which subsequent confirmatory or supplemental assays are automatically initiated following an initial positive result, has emerged as a promising strategy to improve diagnostic efficiency and care linkage in infectious diseases. For example, reflex testing for hepatitis C virus (HCV) RNA following anti-HCV positivity has increased diagnosis and reduced loss to follow-up, particularly among key populations such as people who inject drugs.^9^^,^^10^ The World Health Organization (WHO) now recommends reflex testing as a strategy to promote uptake of HCV RNA and also HBV DNA testing following positive serological screening with HCV antibody and HBsAg, respectively.^5^^,^^11^^,^^12^ Similar principles can be applied to HDV testing, where reflex strategies offer potential to streamline diagnosis, improve care cascades, and support access to earlier treatment initiation, and in particular emerging novel therapies for Delta infection.^5^

Two reflex strategies are relevant in the context of HDV: (1) automatic testing for anti-HDV antibody in all HBsAg-positive individuals and (2) automatic HDV RNA testing in those who are anti-HDV positive. Currently, reflex testing for anti-HDV can only be implemented through a laboratory-based testing strategy since HDV RDTs are not commercially available to enable clinic-based reflex testing. Despite growing interest and implementation in some clinical settings, the overall adoption of laboratory-based reflex testing for HDV remains limited. Moreover, no systematic review and meta-analysis have been conducted to evaluate its impact on improving the HDV care continuum.

To address this gap, we initially conducted this systematic review and meta-analysis to inform the development of potential recommendations on the use of reflex testing for HDV infection in the 2024 WHO guidelines on hepatitis B care and treatment.^12^ Following the publication of the guidelines, we updated the systematic review and meta-analysis following the publication of several key additional studies. Key outcomes assessed included uptake of anti-HDV and HDV RNA testing, linkage to care for both enhanced surveillance for HCC and assessment for treatment, and turnaround times between key steps.

## Methods

### Definitions of HDV reflex testing strategies

Laboratory-based HDV reflex testing means that if the individual's sample for HBsAg screening in the laboratory is positive, then the same or a duplicate specimen is automatically used for a prompt reflex laboratory-based HDV antibody test. Further reflex testing for the presence of HDV RNA can then also be performed for individuals testing positive for HDV antibody (Fig. 2). This means there is a single clinical encounter and blood draw for an initial HBsAg test, after which reflex anti-HDV and HDV RNA testing can be automatically triggered using the same or duplicate specimen. Such an approach has the potential to expedite diagnosis, reduce loss to follow-up, and improve transition efficiency within the HDV care cascade. This enables healthcare workers to provide results for both HBsAg, HDV antibody and potentially HDV RNA at a subsequent follow-up visit. HDV reflex testing for RNA confirmation can currently only be implemented in laboratory settings, as commercial rapid diagnostic tests (RDTs) suitable for clinic-based reflex testing are not available. Previous studies have suggested that laboratory-based reflex testing approach substantially increases HDV antibody case finding and uptake of HDV RNA, while reducing liver disease complications and associated cost.^13^^,^^14^

**Fig. 1:**
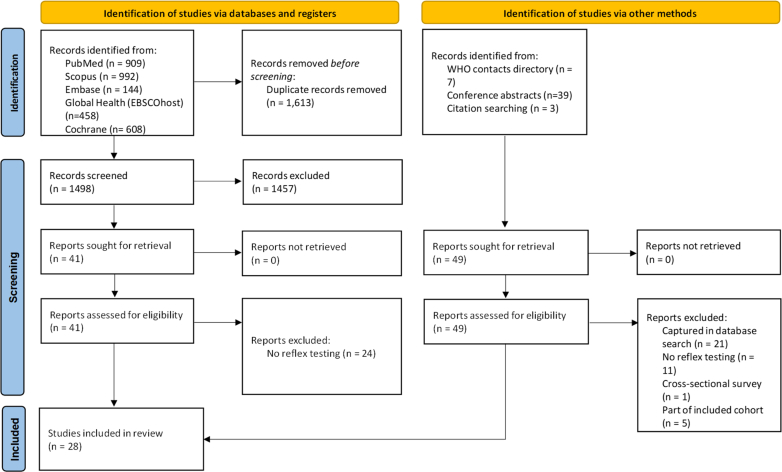
**PRISMA flow diagram of study selection and inclusion for the systematic review.** Abbreviation: WHO, World Health Organization. Conference included: European Association for the Study of the Liver (EASL), American Society for Microbiology (ASM), International Network on Health and Hepatitis in Substance Users (INHSU), American Association for the Study of Liver Diseases (AASLD), Asian Pacific Association for the Study of the Liver (APASL), International Viral Hepatitis Elimination Meeting (IVHEM) and the Conference on Liver Disease in Africa (COLDA).

**Fig. 2:**
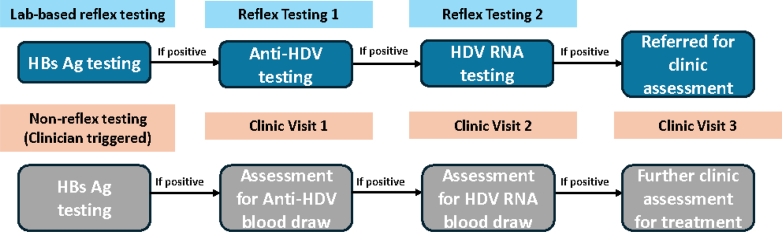
**Schematic overview of laboratory-based HDV reflex testing (top, blue) compared with standard non-reflex testing (bottom, grey). Reflex testing refers to the automatic sequential testing of anti-HDV and HDV RNA following a positive HBsAg result, whereas non-reflex testing requires separate clinical ordering and patient follow-up**.

### Search strategy and selection criteria

This systematic review and meta-analysis were conducted in accordance with PRISMA guidelines.^15^ We searched five electronic databases — PubMed, Embase, Scopus, Cochrane, and Global Health (EBSCOhost) from inception to June, 2025. We included observational studies and randomized controlled trials that evaluated reflex testing strategies for HDV, with or without a non-reflex comparator arm, and reported at least one outcome across the HDV testing and care cascade. We also searched abstracts from seven major international conferences between 2019 and 2024, including the European Association for the Study of the Liver (EASL), American Society for Microbiology (ASM), International Network on Health and Hepatitis in Substance Users (INHSU), American Association for the Study of Liver Diseases (AASLD), Asian Pacific Association for the Study of the Liver (APASL), International Viral Hepatitis Elimination Meeting (IVHEM) and the Conference on Liver Disease in Africa (COLDA). Additionally, we manually reviewed the reference lists of included studies and relevant reviews to identify other potentially eligible studies. Duplicate reports were excluded.

Two reviewers (YT and FL) independently screened titles, abstracts, and full texts, with disagreements resolved by a third reviewer (WT). Studies were included if they evaluated reflex HDV testing strategies and reported data on at least one of the following outcomes: uptake of anti-HDV antibody testing, uptake of HDV RNA testing, linkage to care, and treatment assessment or initiation, and turnaround times between the different steps. No restrictions were applied based on language, geographical region, or population. For conference abstracts, corresponding authors were contacted to verify the operational definition of reflex testing and to confirm key quantitative data. Clarifications or Supplementary Data provided by several authors were incorporated after cross-checking for consistency with the reported results. Study settings were categorized as (1) Hospital-based setting, (2) Laboratory-based setting, (3) Community-based setting, or (4) Mixed setting (where there were multiple settings captured in the same study).

### Data analysis

Two reviewers (FL and YT) independently extracted data on the study setting, population type, WHO region, study design, publication type, age distribution, and gender distribution. Studies were included regardless of whether they had a comparator group (non-reflex testing). Disagreements were resolved through discussion with a third researcher (WT).

Six key outcomes across the cascade of care were evaluated: (1) uptake of anti-HDV testing (number of individuals tested for anti-HDV divided by the number of individuals who were HBsAg-positive), (2) anti-HDV positivity (number of individuals testing positive for anti-HDV divided by the number of individuals tested for anti-HDV), (3) uptake of HDV RNA testing (number of individuals tested for HDV RNA divided by the number who were anti-HDV positive) (4) HDV RNA positive (number of individuals with detectable HDV RNA divided by the number tested for HDV RNA) (5) linkage to care (number of individuals referred to HDV services among those who tested positive for HDV RNA) and (6) turnaround time between these key steps, where available. Meta-analysis was conducted using a random-effects model in R software, (Version 4.5.0) employing the *meta* and *metafor* packages. For each outcome, pooled risk ratios (RRs) and corresponding 95% confidence intervals (CIs) were derived using the DerSimonian–Laird method; when event counts were zero in one arm, a continuity correction of 0.5 was applied. Between-group effects were summarized as absolute proportion differences rather than risk ratios (RRs), to facilitate direct interpretation of service uptake. Statistical heterogeneity was assessed using *Cochran's Q* test and the *I*^*2*^ statistic, with *p < 0.10* and *I*^*2*^ *> 50%* indicating significant heterogeneity. When ≥10 studies were included in a meta-analysis, potential publication bias was evaluated visually using funnel plots and formally tested with Egger's regression.

### Risk of bias and quality assessment

The risk of bias was assessed by two reviewers (FL and YD) using a modified version of the ROBINS-I tool tailored for observational studies reporting binary outcomes.^16^ The quality of evidence was evaluated using the GRADE (Grading of Recommendations, Assessment, Development and Evaluation) framework,^17^^,^^18^ considering the risk of bias, the consistency of results, the directness of the evidence, the precision of the estimates, and the reporting bias.

### Role of the funding source

The study funder had no role in the study design, data collection, data analysis, interpretation of results, or writing of the manuscript.

## Results

### Study characteristics

A total of 3160 records were identified through database searches and other sources. After removal of 1613 duplicates, 1547 unique records were screened by title and abstract, of which 1457 were excluded as not relevant. The remaining 90 full-text articles and abstracts were assessed for eligibility; 62 were excluded as they had not used an HDV reflex testing strategy or there was a lack of relevant outcomes, leaving 28 studies for inclusion in the systematic review and meta-analysis,^13^^,^^14^^,^^19^, ^20^, ^21^, ^22^, ^23^, ^24^, ^25^, ^26^, ^27^, ^28^, ^29^, ^30^, ^31^, ^32^, ^33^, ^34^, ^35^, ^36^, ^37^, ^38^, ^39^, ^40^, ^41^, ^42^, ^43^, ^44^ (Fig. 1). Of the 28 studies, 16 (57.1%) were undertaken in hospital-based settings, four (14.3%) in community-based settings, four (14.3%) in a mixture of community and hospital settings (includes 1 harm reduction site), and nine (32.1%) in laboratory-based settings. Five studies (17.9%) were conducted in low- and middle-income countries (LMICs), while 23 (82.1%) were from high-income countries (HICs) of which the majority. Twenty (71.4%) were from the European region. Nine studies (31.0%) included a non-reflex testing comparison group (Table 1, Supplementary Table S1).

**Table 1:** Summary of characteristics of the 28 included studies.

Study characteristics	Overall, n (%)
**Number of studies**	28
**Studies from HICs (from World Bank 2021)**	23 (82)
**Studies from LMICs (from World Bank 2021)**^a^	5 (18)
**WHO region**^b^	
European Region	20^c^ (71)
Region of the Americas	5^c^ (18)
African Region	3 (11)
Eastern Mediterranean Region	1 (4)
**Study design**	
Retrospective cohort	4 (14)
Prospective cohort	20 (71)
Mixed cohort^d^	1 (4)
Cross-section observational	3 (11)
**Study settings**	
Hospital-based settings	16^e^ (57)
Laboratory-based settings	9^e^ (32)
Community-based settings	4 (14)
Mixed settings	4 (14)
**N studies with non-reflex testing comparator arms**	9 (32)
**Cascade outcomes available for the 28 HDV reflex testing studies**	
Uptake of Anti-HDV antibody testing	28 (100)
Uptake of HDV RNA testing	10 (36)
Linkage to care among HDV RNA positive	3 (11)
Initiation of HDV-specific therapy among HDV RNA positive	3 (11)
**Anti-HDVprevalence among those HBsAg positive(95% confidence interval) (x studies)**	6.2% (4.9–7.9%)
**HDV RNA prevalence in those anti-HDV positive (95% confidence interval) (17 studies)**	45.5% (34.7–56.6%)
**Turnaroundtime data availablefromHBs Ag to Anti-HDV test**	9 (32)

Study countries included: Spain (9), United States (US) (4), France (5), United Kingdom (UK) (2), Egypt (1), Angola (1), Italy (1), Israel (1), Cameroon (1), Nigeria (1), Brazil (1), Austria (1).

a LMICS: Lower-middle-income countries as classified by the World Bank in 2021 – for this review, the LMICs where studies took place were: Angola, Nigeria, Egypt, Cameroon.

b WHO regions are categorized according to the World Health Organization’s regional classification system.

c One study (Hunt, 2023) included two countries: America and Spain.

d Includes both the retrospective phase and the prospective phase.

e One study (Carey, 2023) have 2 hospital-based setting and 1 laboratory-based setting.

### Pooled uptake across the HBV care cascade

Of the 28 studies evaluating reflex testing in 24,796 HBsAg-positive individuals, 22,816 (97.1%, 95% CI: 93.9–98.7) had anti-HDV antibody testing, of whom 1407 (7.2%, 95% CI: 5.8–8.9) tested positive. Of these anti-HDV-positive individuals, 1162 (95.3%, 95% CI: 90.1–97.8) also had HDV RNA testing, and 476 (43.2%, 95% CI: 33.2–53.8) were HDV RNA positive. Linkage to care was reported in only three studies, and all 6 HDV RNA-positive individuals were referred for care (100.0%, 95% CI: 54.1–100.0).

### Reflex testing versus non-reflex testing

Nine studies compared reflex and non-reflex testing approaches. In the reflex arm, 13,871 HBsAg-positive individuals underwent anti-HDV testing (98.0%, 95% CI: 90.9–99.6), and this included data from one abstract that did not report the number of HBsAg-positive individuals. Among the 13,292 tested for anti-HDV antibody, 770 (5.4%, 95% CI: 4.5–6.4) were positive. Of these 770, 598 (99.2%, 95% CI: 93.7–99.9) were tested for HDV RNA, and 260 (41.4%, 95% CI: 10.6–72.2) were RNA positive. In contrast, in the non-reflex arm, 5972 of 24,731 HBsAg-positive individuals were tested for anti-HDV (41.9%, 95% CI: 24.1–57.8), of which 401 (8.1%, 95% CI: 6.0–10.2) were anti-HDV positive. Among these, 233 (36.9%, 95% CI: 14.7–66.4) were tested for HDV RNA.

**Fig. 3:**
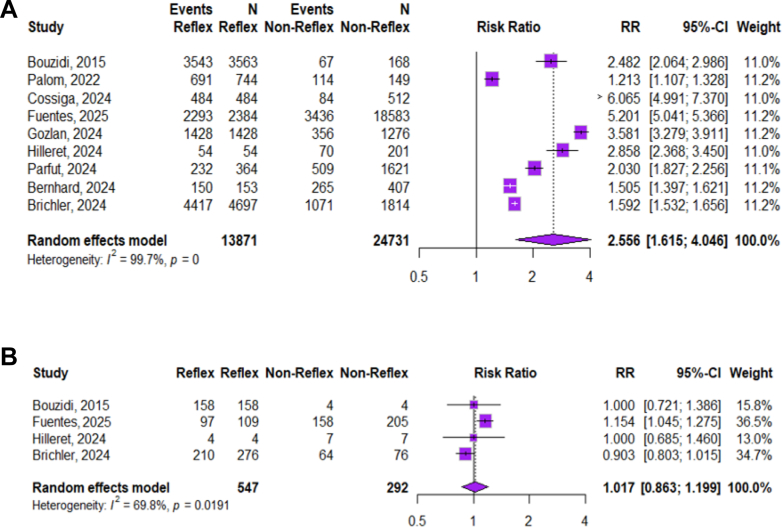
**Meta-analysis of Reflex Versus Non-Reflex Testing Strategies for HDV Detection.** (A) Risk ratios (RRs) for **HDV antibody** testing among HBsAg-positive individuals comparing reflex versus non-reflex testing strategies. (B) Risk ratios (RRs) for **HDV RNA** testing among anti-HDV–positive individuals comparing reflex versus non-reflex testing strategies.

Reflex testing was associated with a substantially higher uptake of anti-HDV testing compared with non-reflex testing approaches, with an absolute pooled proportion difference of **+**32% (95% CI: +18 to +45). There was no significant difference between groups in anti-HDV seropositivity (−3%, 95% CI: −9 to +2) or in HDV RNA positivity among those tested (−4%, 95% CI: −11 to +3). Among anti-HDV–positive individuals, the uptake of HDV RNA testing was also much higher with reflex testing, with a pooled absolute proportion difference of +55.3% (95% CI +26.1% to +84.5%). Sensitivity analyses excluding studies reporting zero uptake in the non-reflex arm produced a pooled absolute difference of +2.6% (95% CI −10.5 to +15.8%), indicating that the overall findings were not driven solely by studies with extreme between-arm differences (Table 3, Fig. 3, and Supplementary Figures S2–S3).

**Table 3:** Summary of outcomes across HDV cascade of care for the 28 HDV reflex testing studies.

First author	HBsAg positive	Anti-HDV tested	Anti-HDV positive	HDV RNA tested	HDV RNA positive	Linkage to care^a^	Initiated HDV treatment
**Hospital-based setting**							
Elzefzafy, 2021	763/204,749 (0.4%)	631/763 (82.7%)	22/631 (3.5%)	22/22 (100.0%)	8/22 (36.4%)		
Brichler, 2022	3198/84,671 (3.8%)	2886/3198 (90.2%)	172/2886 (6.0%)	149/172 (61.1%)	91/149 (61.6%)		
Cobo, 2022	20/2555 (0.8%)	20/20 (100.0%)	3/20 (15.0%)	3/3 (100.0%)	1/3 (33.3%)	1/1 (100.0%)	
Munoz, 2022	276/11,276 (2.4%)	255/276 (92.4%)	19/255 (7.5%)	19/19 (100.0%)	4/19 (21.1%)	4/4 (100.0%)	
Fuentes, 2025		2293/2384 (96.2%)	109/2293 (4.8%)	97/109 (89.0%)	30/97 (31.0%)		
Butí, 2023	159/25,279 (0.6%)		6/159 (3.8%)^c^			73%^c^	
Gonzalez, 2023	3742/2,680,603 (2.8%)	998/3742 (26.7%)	64/998 (6.4%)	64/64 (100.0%)	27/64 (42.2%)		15/27 (55.6%)
Truchi, 2023		68/80 (85.0%)	4/68 (5.9%)				
Wang, 2024	58/9191 (0.6%)	53/58 (91.4%)	3/53 (5.7%)	1/3 (33.3%)	1/1 (100.0%)		
Llanera, 2024	91/17,526 (0.5%)		2/91 (2.2%)				
Parfut, 2024		232/364 (63.7%)	24/232 (10.3%)				
Bernhard, 2024		150/153 (98.0%)	14/150 (9.3%)				
Sobajo, 2022		51/51 (100.0%)	2/51 (3.9%)				
Carey, 2023_Hospital1^b^		238/238 (100%)	17/238 (7.1%)	17/17 (100.0%)	9/12 (52.9%)		
Carey, 2023_Hospital2^b^		322/322 (100%)	23/322 (7.1%)	23/23 (100.0%)	12/23 (52.9%)		
**Pooled estimates (95% CI)**	1.2% (0.5–2.7%)	95.0% (85.9–98.3%)	6.3% (5.2–7.5%)	90.5% (75.6–96.7%)	44.4% (33.8–55.5%)		
**Laboratory-based setting**							
Bouzidi, 2015		3543/3563 (99.4%)	158/3543 (4.5%)	158/158 (100.0%)	32/158 (20.3%)		31/32 (96.9%)
Martínez-Campreciós, 2022	10/93 (10.8%)	10/10 (100.0%)	0/10 (0.0%)				
Palom, 2022		691/744 (92.9%)	56/691 (8.1%)				
Cossiga, 2024	484/33,788 (1.4%)	484/484 (100.0%)	52/484 (10.7%)		26/52 (50%)		
Carey, 2023_Lab1^b^		214/214 (100%)	5/214 (2.3%)	5/5 (100.0%)	1/5 (20.0%)	1/1 (100.0%)	
Hilleret, 2024		54/54 (100%)	4/54 (20.0%)	4/4 (100.0%)	4/4 (100%)		
Gozlan, 2024_site1	1511/151,594 (1.0%)	1511/1511 (100%)	80/1511 (5.3%)	80/80 (100.0%)	60/80 (75.0%)		
Gozlan, 2024_site2			176/2074 (8.5%)	176/176 (100.0%)	19/176 (25.0%)		
Brichler, 2024	6772/459,644 (1.5%)	5749/6772 (84.9%)	364/5749 (6.3%)	285/364 (78.3%)	167/285 (58.6%)		
**Pooled estimates (95% CI)**	2.5% (0.1–42.3%)	99.0% (95.0–99.8%)	6.5% (4.8–8.8%)	97.5% (85.8–99.6%)	41.4% (16.9–71.0%)		
**Community-based setting**							
Picchio, 2023	40/433 (9.2%)	40/40 (100%)	2/40 (5.0%)				
Palom, 2023	9/222 (4.1%)	9/9 (100%)	2/9 (22.2%)	2/2 (100.0%)	2/2 (100%)		
Vasconcelos, 2024	38/430 (8.8%)	32/38 (84.2%)	4/32 (12.5%)				
Zovich, 2024	10/498 (2.0%)	10/10 (100%)	1/10 (10.0%)				
**Pooled estimates (95% CI)**	5.5% (1.8–15.8%)	92.8% (65.4–98.9%)	12.8% (5.1–28.8%)	100.0% (15.8–100.0%)	100.0% (15.8–100.0%)		
**Mixed setting**							
Hunt, 2023_US		326/514 (63.4%)	11/326 (3.4%)	11/11 (100.0%)	4/11 (36.1%)		
Hunt, 2023_Spain		369/371 (99.5%)	27/369 (7.3%)	29/29 (100.0%)	9/27 (33.3%)		
Zahinos, 2024		678/704 (96.3%)					
Ondigui, 2024	133/1992 (6.7%)	133/133 (100%)	42/133 (31.6%)	42/42 (100.0%)	27/42 (64.3%)		
**Pooled estimates (95% CI)**	6.7% (5.6–7.9%)	97.2% (3.2–100.0%)	10.1% (0.4–74.0%)	98.0% (90.2%–99.6%)	46.5% (13.1–83.4%)		
**Pooled estimates (95% CI)**	**2.3% (1.2**–**4.2%)**	**96.8% (93.4**–**98.5%)**	**7.1% (5.8**–**8.8%)**	**94.7% (88.9**–**97.5%)**	**43.9% (33.9**–**54.5%)**	**100.0% (54.1%**–**100.0%)**	

Pooled RRs were derived using random-effects meta-analysis (DerSimonian–Laird method) implemented in the *meta* and *metafor* packages in *R* (version 4.5.0). Where necessary, a continuity correction of 0.5 was applied to zero-cell studies.

a All the HDV-positive patients were linked to a first visit with a specialist post-diagnosis.

b Multiple cohorts from the same study (‘liver specialist cohort 2021’, ‘liver specialist cohort 2022’, ‘community reflex cohort’).

c The denominators of the numbers not given in square brackets were taken directly from the literature, while the numbers in square brackets uniformly used the previous step in cascade as the denominator for quantitative analysis.

### Subgroup analyses

We conducted an additional subgroup analysis of reflex HDV testing across different study settings (Table 2).

**Table 2:** Direct comparison of outcomes across HDV cascade of care for reflex versus non-reflex testing in 9 studies.

Author	Study Arm	Anti-HDV tested	Anti-HDV positive	HDV RNA tested	HDV RNA positive	Initiated treatment
**Hospital-based setting**						
Parfut, 2024	Non-reflex	509/1621 (31.4%)	32/509 (6.3%)			
Parfut, 2024	Reflex	232/364 (63.7%)	24/232 (10.3%)			
Bernhard, 2024	Non-reflex	265/407 (65.1%)	14/265 (5.3%)			
Bernhard, 2024	Reflex	150/153 (98.0%)	14/150 (9.3%)			
Fuentes, 2025	Non-reflex	3436/18,583 (18.5%)	205/3436 (6.0%)	158/205 (77.1%)	69/158 (43.7%)	
Fuentes, 2025	Reflex	2293/2384 (96.2%)	109/2293 (4.8%)	97/109 (89.0%)	30/97 (31.0%)	
**Laboratory-based setting**						
Bouzidi, 2015	Non-reflex	67/168 (39.9%)	4/67 (6.0%)	4/4 (100.0%)	2/4 (50.0%)	
Bouzidi, 2015	Reflex	3543/3563 (99.4%)	158/3543 (4.5%)	158/158 (100.0%)	32/158 (20.3%)	31/32 (96.9%)
Palom, 2022	Non-reflex	114/1492 (7.6%)	11/114 (9.6%)			
Palom, 2022	Single-Reflex	691/744 (92.9%)	56/691 (8.1%)			
Brichler, 2024	Non-Reflex	1071/1814 (59.0)	76/1071 (7.1%)	64/76 (84.2%)	35/64 (54.7%)	
Brichler, 2024	Reflex	4417/4897 (90.2%)	276/4417 (6.2%)	210/276 (76.1%)	109/210 (51.9%)	
Cossiga, 2024	Non-reflex	84/512 (16.4%)	14/84 (16.7%)	14/14 (100.0%)		
Cossiga, 2024	Single-Reflex	484/484 (100.0%)	52/484 (10.7%)	52/52 (100.0%)	26/52 (50%)	
Gozlan, 2024	Non-reflex	356/1276 (27.9%)	38/356 (10.7%)			
Gozlan, 2024	Reflex	1428/1428 (100.0%)	77/1428 (5.4%)	77/77 (100.0%)	59/77 (76.6%)	
Hilleret, 2024	Non-reflex	70/201 (34.8%)	7/70 (10.0%)	7/7 (100.0%)	6/7 (85.7%)	
Hilleret, 2024	Reflex	54/54 (100%)	4/54 (20.0%)	4/4 (100.0%)	4/4 (100%)	
**Risk Ratio (95% CI)**		**2.56 (1.62–4.05)**	**0.85 (0.66–1.08)**	**1.02 (0.86–1.20)**	**0.85 (0.63–1.16)**	

In hospital-based settings, HBsAg positivity was low at 1.2% (0.5–2.7%), but both anti-HDV and HDV RNA testing had high uptake, at 95.7% (87.1–98.6%) and 92.1% (78.2–97.4%), respectively. Among those tested, 7.2% were anti-HDV positive and 43.6% were RNA positive. In community-based settings, HBsAg prevalence was higher at 5.5% (1.8–15.8%). Anti-HDV testing uptake reached 92.8% (65.4–98.9%), and RNA testing was completed in all anti-HDV positive cases. Both anti-HDV and RNA positivity rates were high, at 12.8% and 100.0%, though estimates were imprecise due to small sample sizes. Additional outcome-specific forest plots and subgroup analyses are provided in Supplementary Figures and Tables.

Mixed-setting studies reported similar findings, with anti-HDV and RNA testing uptake at 97.2% and 98.0%, respectively. Anti-HDV positivity was 10.1%, and nearly half (46.5%) of those tested were RNA positive. In laboratory-based settings, testing coverage was near-universal, with 99.0% receiving anti-HDV testing and 97.5% undergoing RNA confirmation. Positivity rates were 6.5% for anti-HDV and 41.4% for HDV RNA.

### Turnaround time

Nine studies (31.0%) reported various turnaround times from testing to results, and four studies also specifically reported turnaround times from HDV RNA testing to results being available, but there were no internal comparators. In each, anti-HDV testing occurred immediately following HBsAg detection (0-day delay), with one study^27^ reported a turnaround time of 20 min. HDV RNA sample collection was also immediate. One study reported a processing time of 1 day from RNA sample collection to results availability,^29^ another study reported a 14-day interval from the time of RNA testing to when results became available.^30^ and a further study reported a mean interval of 26 days (SD 10.5) from initial HBsAg serological testing to treatment initiation.^33^ Because of inconsistent and incomplete reporting across studies, pooled quantitative estimates of turnaround time were not possible, and these findings were therefore summarized descriptively. Together, these findings indicate that reflex testing can accelerate progression through the diagnostic cascade.

### Heterogeneity and publication bias

Considerable heterogeneity was observed across several pooled analyses (I^2^ values often exceeding 80%), reflecting differences in study populations, testing algorithms, and health-care settings. Subgroup analyses by study setting (hospital-, laboratory-, and community-based) partially accounted for this variability. Funnel plots for the main outcomes (anti-HDV antibody testing and HDV RNA testing) showed no marked asymmetry (Supplementary Table).

### Certainty of evidence

Of the 28 studies included in the meta-analysis, 14 were assessed as having a low risk of bias, 7 as moderate, and 7 as high. The most frequent source of bias was in the measurement of outcomes. Fourteen studies did not clearly report whether the tools or procedures used to assess reflex testing uptake, turnaround time, or linkage to care were standardized. Overall, the certainty of evidence was rated as low due to the predominance of observational study designs and demonstrated inconsistencies in reporting key outcomes and methodological details. The inconsistencies included incomplete descriptions of participant selection criteria, lack of clarity regarding the timing and procedures for reflex testing, and insufficient reporting of outcome measures such as uptake rates, turnaround times, and linkage to care. Such variability limited our ability to assess study quality and compare results across studies.

## Discussion

Improving the detection and diagnosis of HDV coinfection among individuals with chronic hepatitis B infection remains a global public health priority. Despite well-established international recommendations advocating anti-HDV testing for all HBsAg-positive individuals, real-world uptake remains low, often due to fragmented workflows and missed clinical opportunities for reflex testing.^45^ This study extends the existing literature by systematically synthesizing evidence on the impact of reflex HDV testing on both anti-HDV and HDV RNA uptake. We found that reflex testing substantially increases uptake of both anti-HDV antibody and HDV RNA testing across diverse populations and settings. Compared with non-reflex strategies, reflex protocols more than doubled the likelihood of completing anti-HDV testing and markedly increased HDV RNA testing among anti-HDV-positive individuals. Reflex workflows also facilitated same-day anti-HDV testing and HDV RNA sample collection, highlighting their potential to expedite diagnosis and streamline linkage to care.

Our findings demonstrate that reflex testing is effective across diverse program types, including hospital-based and community-based programs, and across studies involving people who inject drugs and therefore has the potential for wide application and scale-up. Reflex workflows substantially increase uptake of both anti-HDV and HDV RNA testing, facilitate same-day sample collection, and streamline linkage to care. National and global surveys indicate growing adoption of reflex testing in laboratory networks, with many facilities either implementing or planning these workflows, reflecting increasing momentum toward wider adoption.^46^ Although anti-HDV screening requires only standard serological testing, HDV RNA testing is more complex, relying on access to real-time PCR platforms, clear protocols, and trained laboratory personnel, which can pose challenges in low-resource settings.^47^ Nevertheless, ongoing investments in molecular platforms through hepatitis B, hepatitis C, and HIV programs are expanding testing capacity and enabling reflex HDV testing as part of routine diagnostic pathways. Integrating HDV reflex testing into existing hepatitis B care programs can lead to earlier diagnosis and allow faster referral for treatment evaluation, underscoring its scalability and potential impact on strengthening case finding and improving health outcomes.

This review has several limitations. First, the number of included studies was limited, with only nine directly comparing reflex versus non-reflex strategies, which may limit the generalizability of effect estimates. Second, several included studies were available only as conference abstracts or presentations, which provided limited methodological detail and may introduce uncertainty in data interpretation. Third, only 9 studies reported on turnaround time across the HDV testing and care cascade, and none of these had a non-reflex comparator arm, which precluded a comprehensive analysis of how reflex testing contributes to timely diagnosis of HDV coinfection. Fourth, due to the heterogeneity in study design and limited data availability, formal meta-analyses were not feasible for some outcomes, and a descriptive synthesis approach was therefore applied. Fifth, although study settings were categorized by WHO region, the majority of studies were conducted in the European Region, which limited our ability to perform regional subgroup analyses. Finally, heterogeneity in testing protocols and patient populations may have contributed to variability in outcomes, although sensitivity analyses confirmed the overall robustness of findings.

Our findings support the recent 2024 WHO guideline recommendations on testing for Delta infection,^12^^,^^48^ which emphasize reflex anti-HDV antibody testing following a positive HBsAg test result and reflex HDV RNA testing (where available) following a positive anti-HDV antibody result as additional strategies to promote diagnosis (conditional recommendation, low-certainty evidence). Integrating double HDV reflex testing into routine HBV clinical and laboratory workflows should now therefore be considered standard practice. To support implementation, countries should adopt standardized reflex testing protocols, ensure test costs are covered under public health insurance, and invest in laboratory infrastructure to enable timely molecular confirmation.^49^

Recent analyses further support the economic and practical feasibility of reflex or universal HDV screening. A U.S. cost-effectiveness study found that routine HDV screening among HBsAg-positive individuals is cost-saving in moderate-to-high prevalence contexts,^50^ but an implementation study by Mageras and colleagues identified key logistical and system-level barriers to adoption of reflex testing within U.S. healthcare settings.^51^

From a research perspective, future studies should investigate real-world implementation barriers, including variability in laboratory capacity, workforce training, and test availability across settings. Further evaluation is needed to assess cost-effectiveness of integrated reflex testing approach that includes HIV, hepatitis B and C or programmes. Understanding these challenges will be critical to optimizing uptake, especially in low- and middle-income countries, where infrastructure and resources remain limited.

## Contributors

PE conceptualized the study. PE, WT, and YT designed the study. FL and YT reviewed and assessed the studies for inclusion. FL and YT accessed and extracted data. FL, YD, CF and YT performed the risk of bias assessment. YT and WT verified the underlying data. FL, WT, and RC contributed to the GRADE analysis. YT and WT carried out analyses and produced the first draft of the manuscript. All authors contributed to the interpretation of data and revising the manuscript critically for important intellectual content. All authors approved the final manuscript and take responsibility for the decision to submit for publication.

## Data sharing statement

Aggregate, rather than individual-level, data were included in these analyses from published manuscripts and conference publications, which are publicly available. For aggregate data taken from the grey literature that are not publicly available, please contact the corresponding author.

## Declaration of interests

The authors declare no competing interests. Roger Chou reports receiving a consulting fee for serving as a WHO methodologist for the HBV guideline for which this review was commissioned.
