# Supplementary material for: Double reflex testing for anti-HDV antibody following an HBsAg-positive test result and HDV RNA in those anti-HDV positive: a global systematic review and meta-analysis
Source: eClinicalMedicine. 2026 Jan 5;91:103708. doi: 10.1016/j.eclinm.2025.103708 (PMC12809736; doi:10.1016/j.eclinm.2025.103708)
Supplement: Supplementary Figures and Tables [file mmc1.docx]

**Supplementary Material:**

**Table 1. Characteristics of 28 included studies**

|  | **Study population** | **Study Setting** | **City or region,**  **country** | **Study Period** | **Study Design** | **Study Source** | **Number of**  **study arms** | **Reflex testing approach** | | **Number of**  **population** | **Risk of bias** |
| --- | --- | --- | --- | --- | --- | --- | --- | --- | --- | --- | --- |
| **Bouzidi, 2015** | HBV patients | Tertiary hospitals | London, UK | Non-reflex: 2012/6-2012/8;  Reflex: 2001-2012 | Cross-sectional study | Main database search | 2 | Double-reflex | Non-reflex: 168; Reflex: 3563 | | Low |
| **Elzefzafy, 2021** | HBV patients | Outpatient clinics | Nile Delta, Egypt | 2016/1-2018/8 | Prospective | Main database search | 1 | Double-reflex | 204749 | | Low |
| **Martínez-Campreciós, 2021** | Hospital  outpatients | Hospital’s outpatient  clinic | Cubal, Angola | 2019/12 | Prospective | Main database search | 1 | Single-reflex | 93 | | Low |
| **Palom, 2022** | HBsAg-positive | Central laboratory | Barcelona, Spain | Non-reflex: 2018/1-2020/12;  Reflex: 2021/1-2021/12 | Prospective | Main database search | 2 | Single-reflex | Non-reflex: 1492;Reflex: 744 | | Low |
| **Brichler, 2022** | HBsAg positive | University Hospital | Paris, France | 2012/6-2022/2 | Retrospective | Conference abstract search | 1 | Double-reflex | 84671 | | Low |
| **Cobo, 2022** | Hospital outpatients | Emergency Department | Barcelona, Spain | 2020/4/-2022/8 | Prospective | Conference abstract search | 1 | Double-reflex | 2555 | | Low |
| **Munoz, 2022** | Hospital outpatients | Primary care centers,  treatment units, hospital departments, outpatient hospital and penitentiary center | Valencia, Spain | 2022/4-2022/8 | Prospective | Conference abstract search | 1 | Double-reflex | 11276 | | Low |
| **Cossiga, 2024** | HBsAg positive subjects | University Hospital | Naples, Italy | 2019/1-2022/12 | Prospective | Main database search | 1 | Double-reflex | Non-reflex: 32649; Reflex: 33788 | | Low |
| **Fuentes, 2025** | HBsAg positive patients | Hospitals | Andalusia, Spain | 2022/10-2023/3 | Both Prospective and Retrospective | Main database search | 1 | Double-reflex | Non-reflex: 18583; Reflex: 2384 | | High |
| **Butí, 2023** | Hospital outpatients | Urban hospital | Barcelona, Spain | 2020/2-2023/5 | Prospective | Conference abstract search | 1 | Single-reflex | 25279 | | Some |
| **Carey, 2023** | HBsAg-positive | Specialist viral hepatitis  laboratory | London, UK | 2021/1-2022/12 | Prospective | Conference abstract search | 1 | Double-reflex | 238 | | Some |
| **Gonzalez, 2023** | General population | University hospital  and city laboratories | Paris, France | 2019/1-2022/12 | Retrospective | Conference abstract search | 1 | Double-reflex | 2680603 | | Some |
| **Hunt, 2023** | HBsAg-positive | Hospitals and communityhealth centers | USA; Spain | 2022/4-2023/2 | Prospective | Conference abstract search | 1 | Double-reflex | 68536 | | Some |
| **Picchio, 2023** | General populations | Community | Barcelona,  Spain | 2020/11-2022/01 | Prospective | Main database search | 1 | Single-reflex | 433 | | Some |
| **Truchi, 2023** | HBsAg positive | Virology laboratory of  University Hospital | Nice, France | 2021/1-2021/12 | Prospective | Conference abstract search | 1 | Single-reflex | 80 | | Some |
| **Zahinos, 2024** | HBV patients | Primary healthcare; hospital healthcare and addiction centers and prisons | Barcelona, Spain | 2022/1-2023/11 | Prospective | Conference abstract search | 1 | Single-reflex | 704 | | Some |
| **Wang, 2024** | HBV patients | Community hospital | USA | 2018-2022 | Prospective | Conference abstract search | 1 | Double-reflex | 9191 | | Low |
| **Brichler, 2024** | HBsAg positive | Hospital laboratories | France | 2022/08-2024/04 | Retrospective | Main database search | 1 | Single-reflex | Non-reflex:1814; Reflex: 4697 | | Low |
| **Gozlan, 2024** | HBsAg positive | Maccabi virology  laboratory | Israel | 2021/11/1-2023/10/31 | Prospective | Conference abstract search | 2 | Double-reflex | Non-reflex:144512; Reflex: 151594 | | Some |
| **Hilleret, 2024** | HBsAg positive | Laboratory | Montbonnot-Saint-Martin, France | 2021-2023/2 | Prospective | Main database search | 2 | Double-reflex | Non-reflex: 201; Reflex: 54 | | Some |
| **Llanera, 2024** | Hospital outpatients | Referral hospital | Barcelona, Spain | 2020/2-2022/2 | Prospective | Main database search | 1 | Single-reflex | 17560 | | Some |
| **Ondigui, 2024** | General or mixed population | Tertiary hospital, District  hospitals, and Health  Center | Cameroon | 2019/1-2022/7 | Cross-sectional study | Main database search | 1 | Double-reflex | 1992 | | Low |
| **Palom, 2023** | General or mixed population | Community setting | Barcelona, Spain | 2022/4-2022/11 | Prospective | Main database search | 1 | Double-reflex | 280 | | High |
| **Parfut, 2024** | HBsAg positive | University Hospital | Strasbourg, France | 2017-2022 | Prospective | Main database search | 2 | Single-reflex | Non-reflex: 1621;Reflex: 364 | | Low |
| **Sobajo, 2024** | Hospital outpatients | Tertiary hospitals | Nigeria |  | Cross-sectional study | Main database search | 1 | Single-reflex | 51 | | Low |
| **Vasconcelos, 2024** | General or mixed population | Basic health posts | Brazil | 2022 | Prospective | Main database search | 1 | Single-reflex | 430 | | Low |
| **Zovich, 2024** | People who use drugs (PWUD) | Harm reduction  organization (HRO) | Philadelphia, US | 2023/8-2023/9 | Prospective | Main database search | 1 | Single-reflex | 513 | | Some |
| **Bernhard, 2024** | HBsAg positive | Vienna General Hospital | Vienna, Austria | 2018-2022 | Retrospective | Main database search | 2 | Single-reflex | Non-reflex: 407; Reflex:153 | | Low |

**Table 2. Search strategy and terms**

| Set# |  |
| --- | --- |
| 1 | "Hepatitis B" [Mesh] OR hepatitis-B [tiab] OR “hepatitis B” [tiab] OR “hep b” [tiab] OR hep-b [tiab] OR HBV [tiab] OR HBsAg [tiab] OR “serum hepatitis” [tiab] OR “hepatitis type B” [tiab] |
| 2 | "Hepatitis Delta Virus" [Mesh] OR hepatitis-delta [tiab] OR “hepatitis delta” [tiab] OR “delta virus” [tiab] OR “delta viruses” [tiab] OR HDV [tiab] OR anti-HDV [tiab] OR “hepatitis delta virus” [tiab] OR “hepatitis delta viruses” [tiab] OR “delta agent” [tiab] OR “delta agents” [tiab] OR delta virus [tiab] OR delta viruses [tiab] OR hepatitis-D [tiab] OR “hepatitis D” [tiab] OR “hep d” [tiab] OR hep-d [tiab] |
| 3 | "Point-of-Care Systems" [Mesh] OR "Point-of-Care Testing" [Mesh] OR POCS [tiab] OR POCT [tiab] OR “same-day diagnosis”[tiab] OR “same day diagnosis”[tiab] OR “same-day diagnoses” [tiab] OR “same day diagnoses”[tiab] OR “single visit diagnosis”[tiab] OR “single-visit diagnosis” [tiab] OR “single visit diagnoses”[tiab] OR “single-visit diagnoses” [tiab] OR SVD[tiab] OR ((reflex[tiab] OR remote[tiab] OR rapid[tiab] OR point-of-care [tiab] OR “point of care”[tiab] OR POC[tiab] OR decentralized[tiab] OR decentralization [tiab] OR decentralizes[tiab] OR decentralize[tiab] OR “community based” [tiab] OR community-based[tiab] OR outreach[tiab] OR simplified[tiab] OR near-patient [tiab] OR “near patient”[tiab] OR bedside[tiab] OR on-demand[tiab] OR “on demand” [tiab] OR one-time[tiab] OR one-stop[tiab] OR one-step[tiab] OR cascade [tiab]) AND ("Reagent Kits, Diagnostic"[Mesh] OR "Serologic Tests"[Mesh] OR testing [tiab] OR test[tiab] OR tests[tiab] OR tested[tiab] OR kit[tiab] OR kits[tiab] OR detect* [tiab] OR diagnosis[tiab] OR diagnosed[tiab] OR diagnosing[tiab] OR diagnoses [tiab] OR diagnostic[tiab])) |
| 4 | #1 AND #2 AND #3 |

**Table 3. Time (days) between key steps in the HDV cascade of care for each of the 9 reflex testing studies with time data available.**

| **Author** | **Study settings** | **Ag test to anti-HDV**  **test** | **RNA test to results made available** | **First test to treatment start** |  |
| --- | --- | --- | --- | --- | --- |
| Bouzidi, 2015 | Laboratory-based setting | 0 |  | 1 |  |
| Martinez-Camprecios, 2021 | Laboratory-based setting | 0 |  |  |  |
| Palom, 2022 | Laboratory-based setting | 0 |  |  |  |
| Picchio, 2023 | Community-based setting | 0 |  | 26 (SD: 10.5) |  |
| Zovich, 2024 | Community-based setting | 0 |  |  |  |
| Palom, 2023 | Community-based setting | 0 (20mins) | 0 |  |  |
| Ondigui, 2024 | Mixed setting | 0 | 0 | 14 |  |
| Cobo, 2022 | Hospital-based setting | 0 (sequential) | 0 (sequential) |  |  |
| Munoz, 2022 | Hospital-based setting | 0 (sequential) | 0 (sequential) |  |  |

**Table 4. Summary of Different Settings of the 29 Included Studies**

|  | **Overall**  **(n=2**8**)** | **Hospital-based setting**  **(n=16*)** | | **Laboratory-based setting**  **(n=7)** | | **Community-based setting**  **(n=4)** | **Mixed setting**  **(n=4)** |
| --- | --- | --- | --- | --- | --- | --- | --- |
| **Studies from LMICs^1^**  **(from world Bank 2021)** | 5 | 2 | 1 | | 1 | | 1 |
| **Studies from WHO region** |  |  |  | |  | |  |
| Africa | 3 | 1 | 1 | | 0 | | 1 |
| Americas | 5* | 2 | 0 | | 2 | | 1 |
| Eastern Mediterranean | 1 | 1 | 0 | | 0 | | 0 |
| European | 20* | 9 | 6 | | 2 | | 2 |
| **Studies with non-reflex**  **comparator arms** | 9 | 5 | 0 | | 3 | | 1 |

^1^ LMICS: Lower-middle-income countries as classified by the World Bank in 2021 – for this review, the LMICs where studies took place were: Single-reflex testing (Angola, Nigeria);

Double-reflex testing (Egypt, Cameroon).

*One study (Hunt, 2023) included two countries: America and Spain so counted twice and one study (Carey, 2023) have 2 hospital-based setting and 1 laboratory-based setting.

**
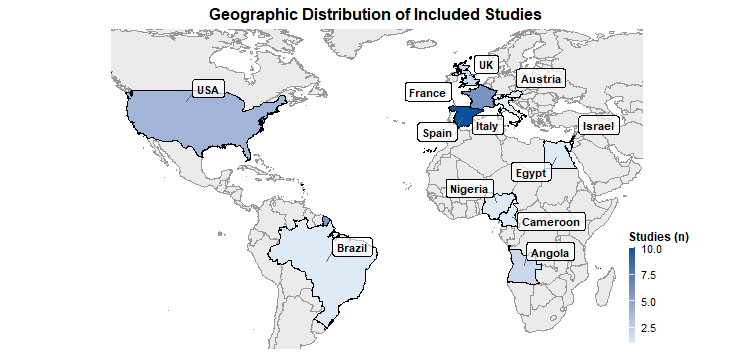
**

**Figure 1. Geographic distribution of countries from 28 studies included within the review (shaded in blue).**

WHO region: **Europe** – Italy (n=1), France (n=6), Israel (n=1), Spain (n=9), UK (n=2), Austria (1). **Africa** – Angola (n=2), Nigeria (n=1), Cameroon (n=1). **Americas** – USA (n=3), Brazil (n=1). **Eastern Mediterranean** – Egypt (n=1).

4 (14%) from lower-middle-income countries, 1 (4%) from upper-middle-income countries, and 23 (82%) from high-income countries.

**Risk of bias**

***METHODS: RISK OF BIAS***

Authors FL and YT both assessed the risk of bias for each study using a previously published and modified risk of bias tool used for observational studies that report binary outcomes based on tools by Hoy et al and the ROBINS-I tool^4-6^. Due to a lack of any RCTs, the question “Was some form of random selection used to select the sample?” was modified to “Was prospective selection used to select the sample?”. Where there was a disagreement between the two overall risk of bias assessments from the initial assessors, a third author, WT, arbitrated.

**Domain of bias:**

Selection bias:

1. Was the study’s target population pre-specified and appropriately chosen to answer the study question(s)?
2. Was the sampling frame a true or close representation of the target population?
3. Was prospective selection used to select the sample? Bias due to missing outcome data:
4. Was the proportion of missing outcome data minimal and were there none/minimal differences between those with and without missing outcome data?

Bias in measurement of the outcome:

1. Were data collected directly from the subjects (as opposed to a proxy?)
2. Was an acceptable case definition used in the study?
3. Was the study instrument that measured the parameter of interest (e.g. proportion of results obtained on the same day) shown to have reliability and validity?
4. Was the same mode of data collection used for all subjects?
5. Was the length of the shortest measurement period appropriate for the parameter of interest?

Bias in analysis:

1. Were the numerator(s) and denominator(s) for the parameter of interest appropriate?

**Overall judgement of risk of bias:**

Low risk: all domains indicated as low risk

Some/medium risk: 1 or no domains have signaling question indicating high risk; OR, at least one signaling question without information.

High risk: 2 or more domains have signaling questions indicating high risk

**Table 5. Risk of bias score for each study**

| **Study** | **1.**  **Target population** | **2.**  **Sampling frame** | **3.**  **Prospective selection** | **4.**  **Missing outcome** | **5.**  **Data from**  **subjects** | **6.**  **Acceptable case**  **definition** | **7.**  **Study instrument** | **8.**  **Same mode of collection** | **9.**  **Appropriate parameter** | **10.**  **Num/den appropriate** | **Overall**  **bias** |
| --- | --- | --- | --- | --- | --- | --- | --- | --- | --- | --- | --- |
| Bouzidi, 2015 | Y | Y | Y | Y | Y | Y | Y | Y | Y | Y | Low |
| Elzefzafy, 2021 | Y | Y | Y | Y | Y | Y | Y | Y | Y | Y | Low |
| Martínez-Campreciós, 2021 | Y | Y | Y | Y | Y | Y | Y | Y | Y | Y | Low |
| Palom, 2022 | Y | Y | Y | Y | Y | Y | Y | Y | Y | Y | Low |
| Brichler, 2022 | Y | Y | Y | Y | Y | Y | Y | Y | Y | Y | Low |
| Cobo, 2022 | Y | Y | Y | Y | Y | Y | Y | Y | Y | Y | Low |
| Munoz, 2022 | Y | Y | Y | Y | Y | Y | Y | Y | Y | Y | Low |
| Cossiga, 2024 | Y | Y | Y | Y | Y | Y | Y | Y | Y | Y | Low |
| Fuentes, 2024 | Y | Y | Y | Y | Y | Y | Y | Y | Y | Y | Low |
| Butí, 2023 | Y | Y | Y | Y | Y | Y | N | Y | Y | Y | Medium |
| Carey, 2023 | Y | Y | Y | N | Y | Y | N | Y | Y | Y | High |
| Gonzalez, 2023 | Y | Y | Y | Y | Y | Y | N | Y | Y | Y | Medium |
| Hunt, 2023 | Y | Y | Y | N | Y | Y | N | Y | Y | Y | High |
| Picchio, 2023 | Y | Y | Y | Y | Y | Y | N | Y | Y | Y | Medium |
| Truchi, 2023 | Y | Y | Y | N | Y | Y | N | Y | Y | Y | High |
| Zahinos, 2024 | Y | Y | Y | N | Y | Y | N | Y | Y | Y | High |
| Wang, 2024 | Y | Y | Y | N | Y | Y | N | Y | Y | Y | High |
| Brichler, 2023 | Y | Y | Y | N | Y | Y | N | Y | Y | Y | High |
| Gozlan, 2024 | Y | Y | Y | N | Y | Y | Y | Y | Y | Y | Medium |
| Hilleret, 2024 | Y | Y | Y | N | Y | Y | Y | Y | Y | Y | Medium |
| Llanera, 2024 | Y | Y | Y | Y | Y | Y | N | Y | Y | Y | Medium |
| Ondigui, 2024 | Y | N | Y | N | Y | Y | Y | Y | Y | Y | High |
| Palom, 2023 | Y | Y | Y | Y | Y | Y | Y | Y | Y | Y | Low |
| Parfut, 2024 | Y | Y | Y | Y | Y | Y | Y | Y | Y | Y | Low |
| Sobajo, 2024 | Y | Y | Y | Y | Y | Y | Y | Y | Y | Y | Low |
| Vasconcelos, 2024 | Y | Y | Y | N | Y | Y | Y | Y | Y | Y | Medium |
| Zovich, 2024 | Y | Y | Y | Y | Y | Y | Y | Y | Y | Y | Low |
| Bernhard, 2024 | Y | Y | Y | Y | Y | Y | Y | Y | Y | Y | Low |

**Table 6. Grading the standard of evidence for services uptake across HDV care cascades using the reflex HDV testing approach.**

| **Outcome** | **Evidence** | **Risk of bias** | **Inconsistency** | **Imprecision** | **Indirectness** | **SOE** | **Main Findings** |  |
| --- | --- | --- | --- | --- | --- | --- | --- | --- |
| Percentage of HBsAg positive among people who have HBsAg test | Sixteen study  arms from observational studies (n=50,870) | Serious risk of bias | No inconsistency | No imprecision | Serious indirectness | Very Low | A pooled rate of 2.3% (1.2-4.2%) |  |
| Percentage of Anti-HDV tested among people who were HBsAg positive | Fourteen study arms from observational studies (n=28,788) | Some risk of bias | No inconsistency | No imprecision | Serious indirectness | Low | A pooled rate of 96.8%  (93.4-98.0%) |  |
| Percentage of Anti-HDV positive among people who have Anti-HDV test | Twenty-eight study arms from observational studies (n=1,808) | Serious risk of bias | No inconsistency | No imprecision | Serious indirectness | Very Low | A pooled rate of 7.1% (5.8-8.8%) |  |
| Percentage of HDV RNA tested among people who were Anti-HDV positive | Thirteen study arms from observational studies (n=1,391) | Serious risk of bias | No inconsistency | No imprecision | Serious indirectness | Low | A pooled rate of 94.7%  (88.9-97.5%) |  |
| Percentage of HDV RNA positive among people who have HDV RNA test | | Twenty-four study arms from observational studies (n=655) | Serious risk of bias | No inconsistency | No imprecision | Serious indirectness | Very Low | A pooled rate of 43.9% (33.9-54.5%) |
| Percentage of people linked for care who were anti-HDV positive | Three study arms from observational studies (n=35) | Serious risk of bias | No inconsistency | No imprecision | Serious indirectness | Very Low | A pooled rate of 100.0%  (99.1%-100.0%) |  |

*The risk of bias was downgraded by one level due to the majority moderate risk of bias (7/8).

** The indirectness was downgraded since only one directly compared reflex with non-reflex, and most findings are primarily based on indirect (across study) comparison.

**Comparison Forest plots:**

**
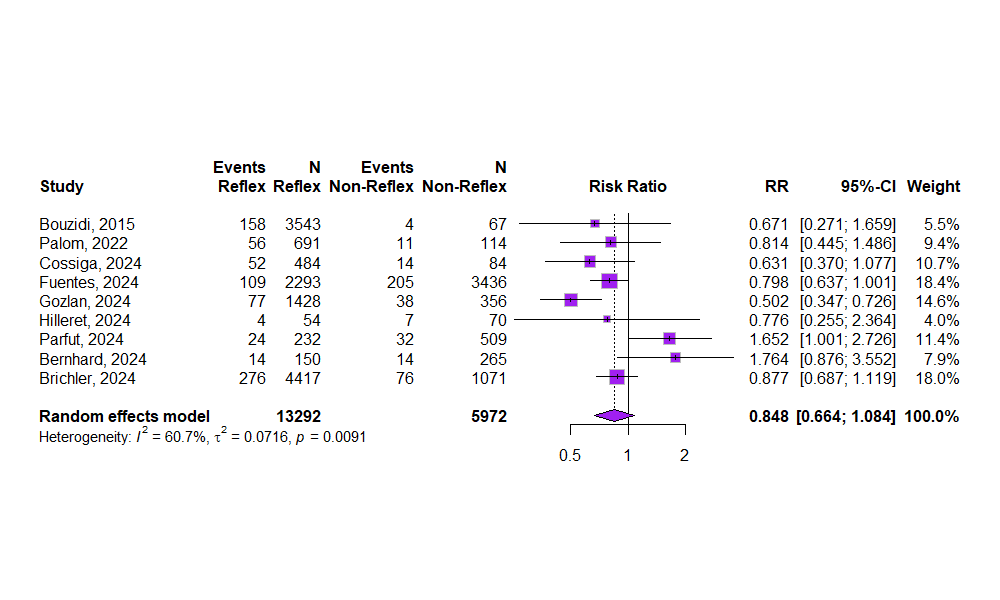
**

**Figure 2. Comparison of Anti-HDV Positivity Rates Between Reflex and Non-Reflex Testing Approaches**

**
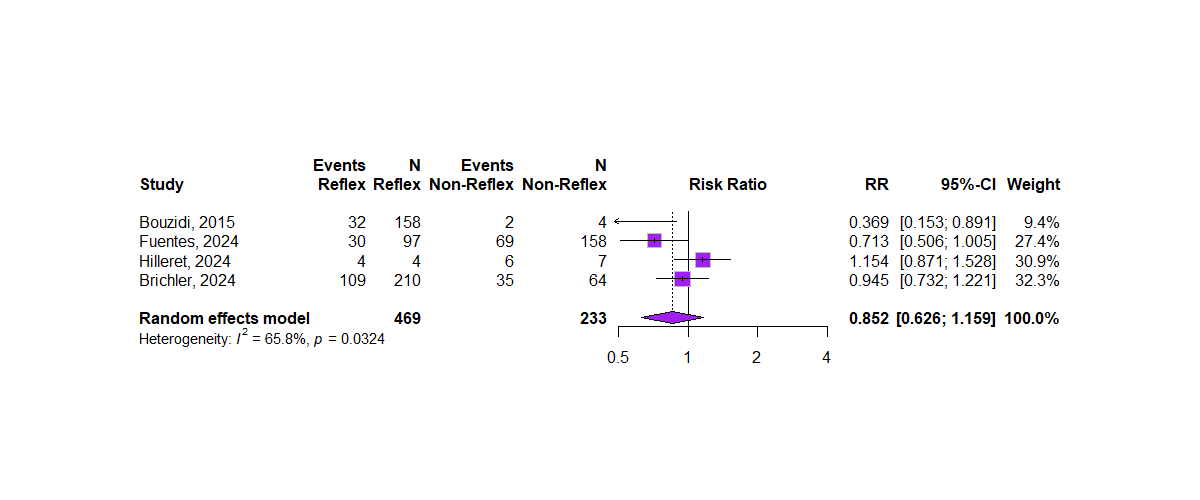
**

**Figure 3. Comparison of HDV RNA Positivity Rates Between Reflex and Non-Reflex Testing Approaches**

**Other Forest Plots:**

**Pooling results for all reflexing testing:**

**a: HBs Ag+ among who tested for HBs Ag**

**
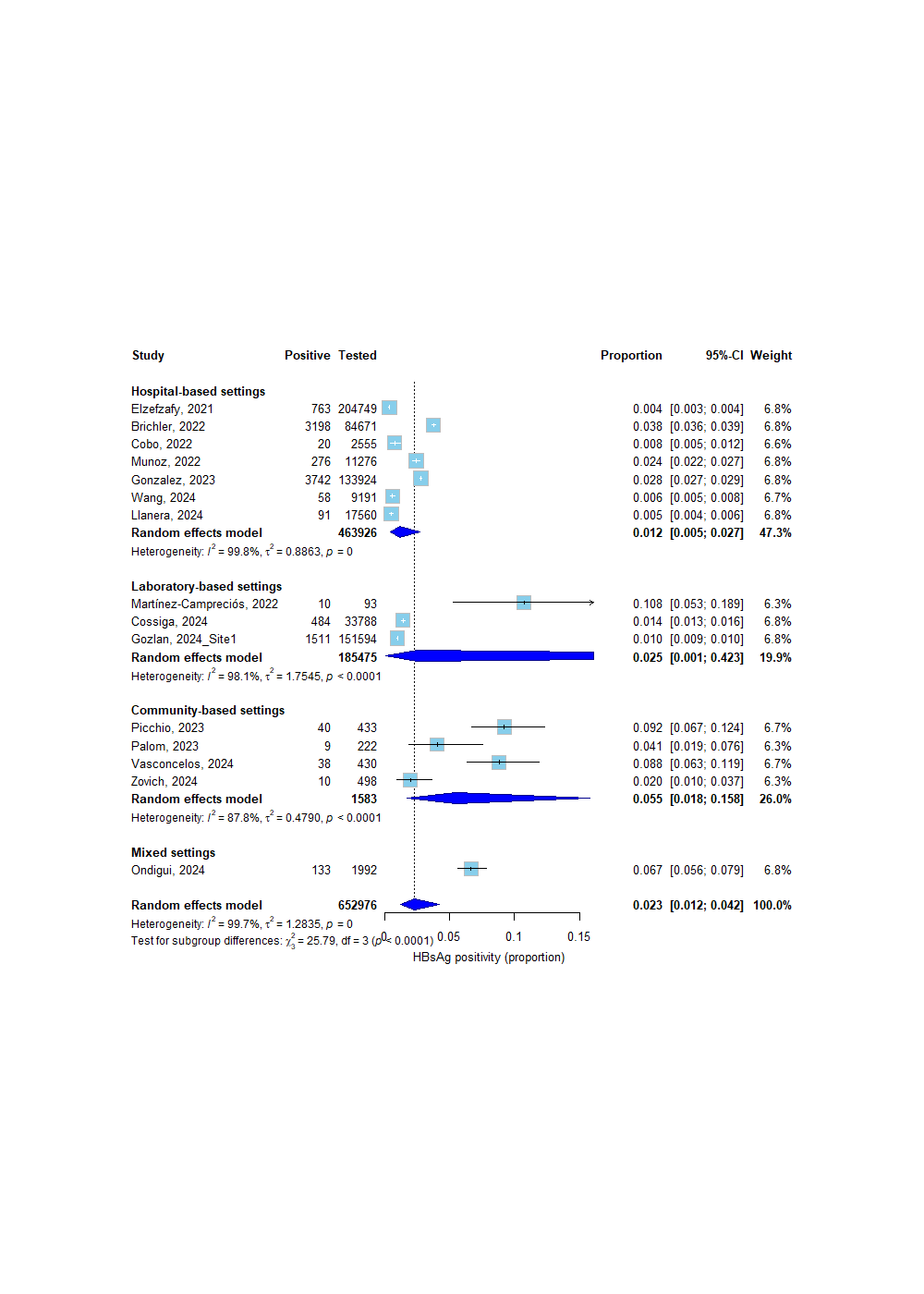
**

**b: Anti-HDV tested among who HBs Ag+**

**
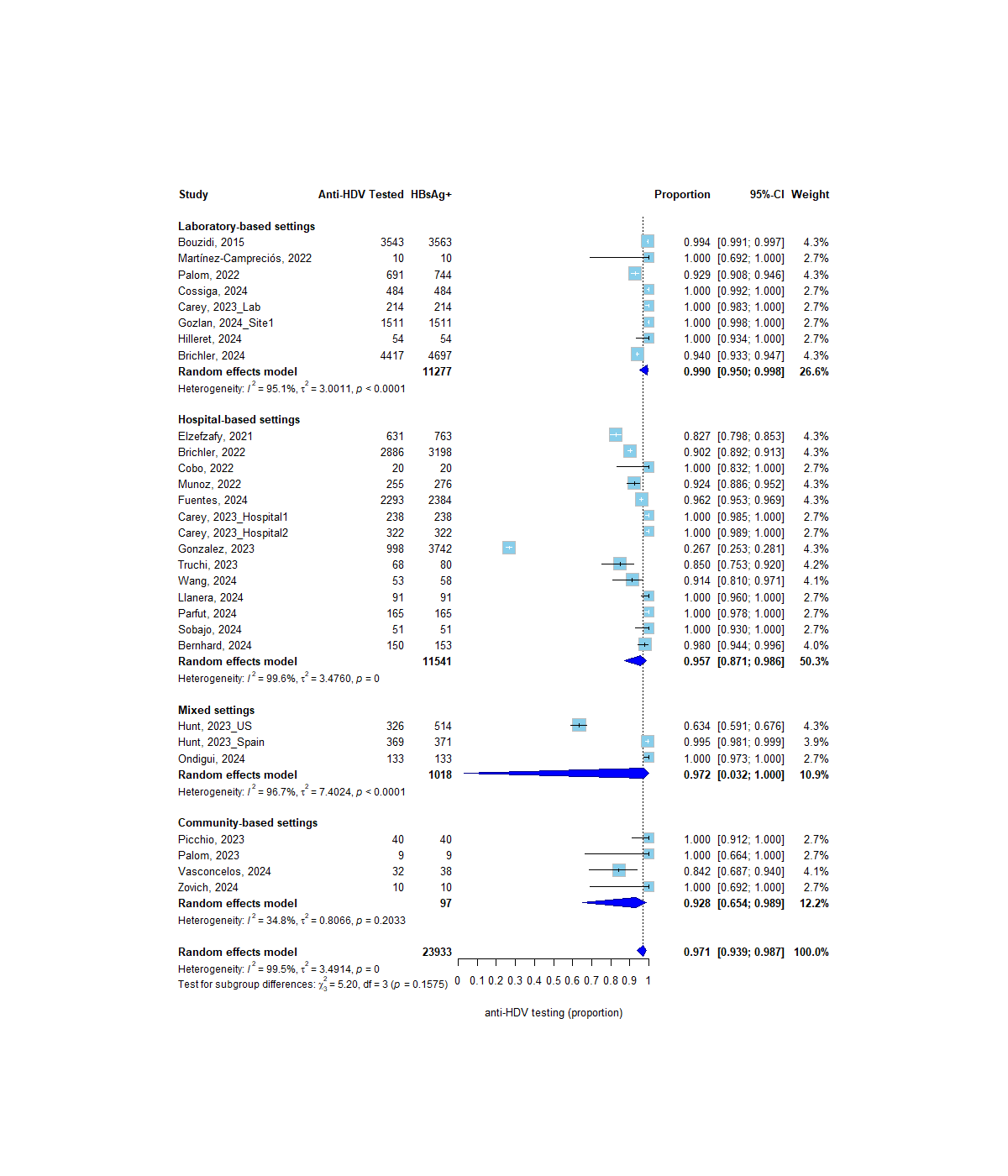
**

**c: Anti-HDV+ among who tested Anti-HDV**

**
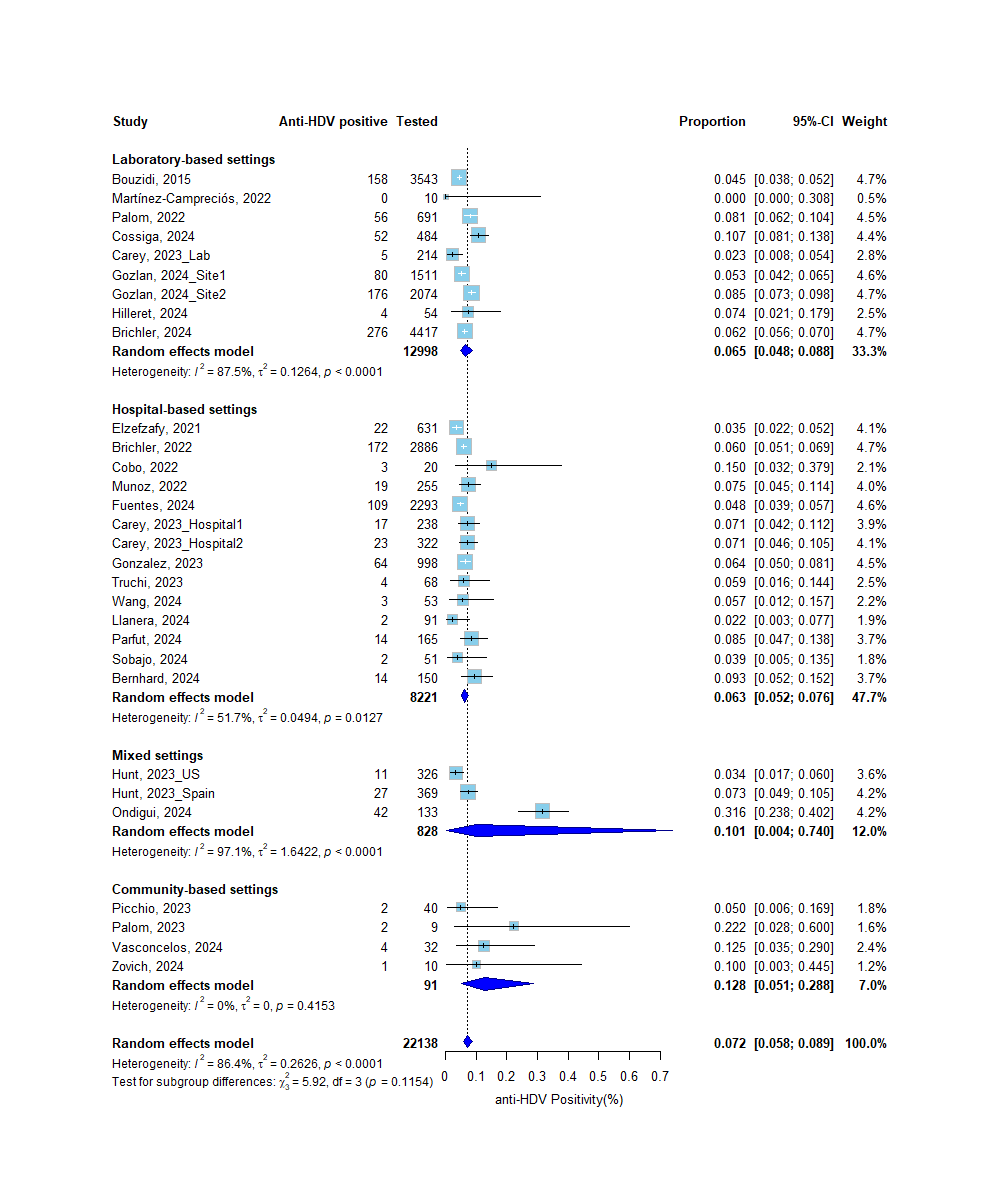
**

**d: HDV RNA tested among who Anti-HDV+**

**
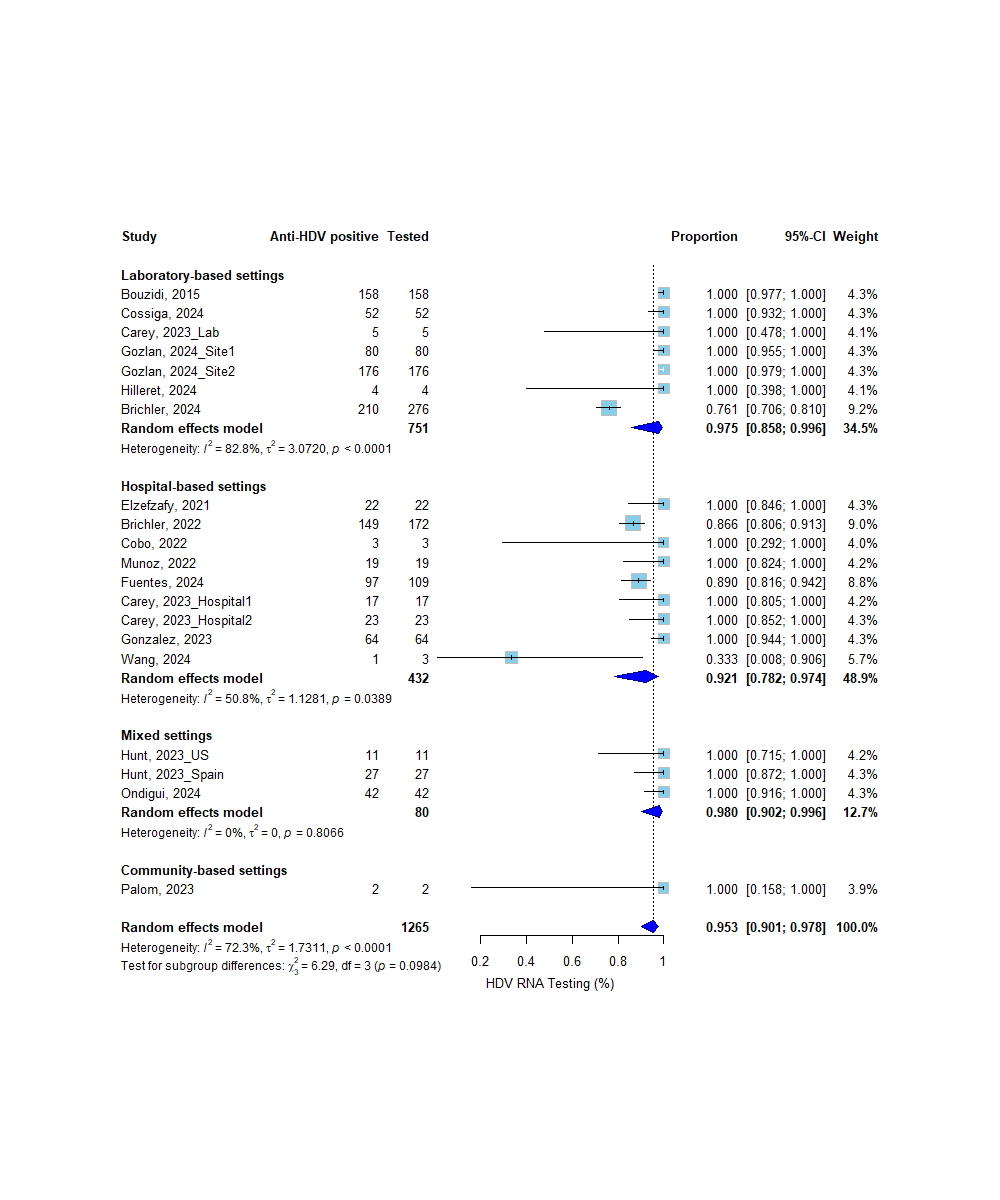
**

**e: HDV RNA positive among who tested for HDV RNA**

**
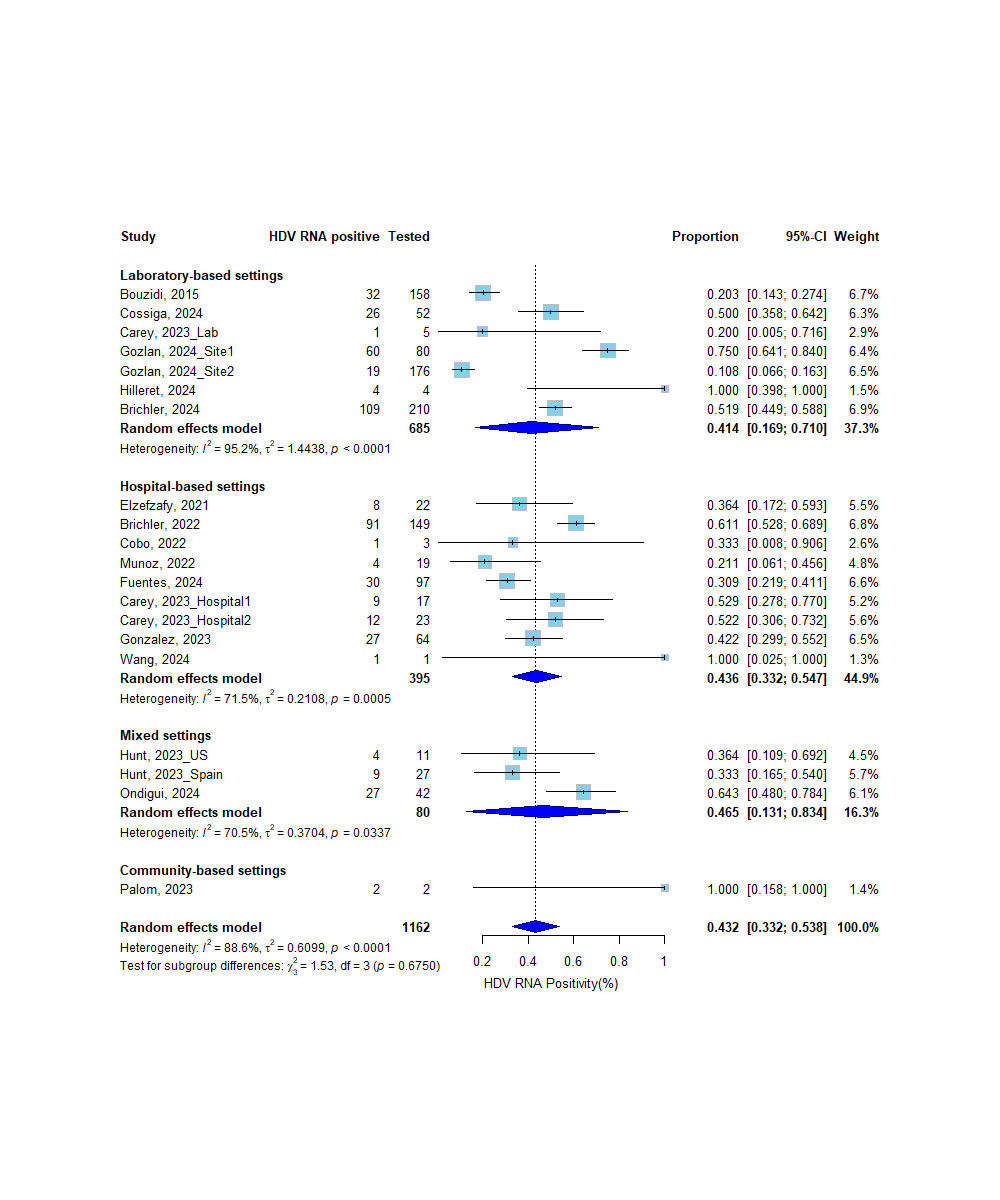
**

**f: Linkage to care among who’s HDV RNA positive**


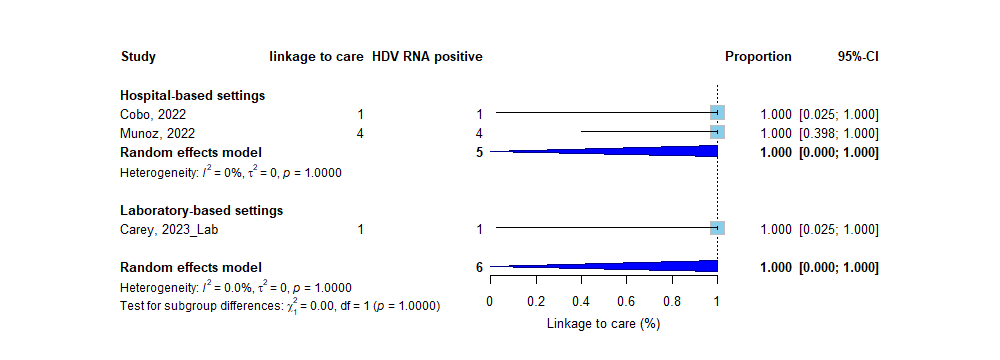


**Pooling results for non-reflex testing:**

**a: Anti-HDV tested among who HBs Ag+**

**
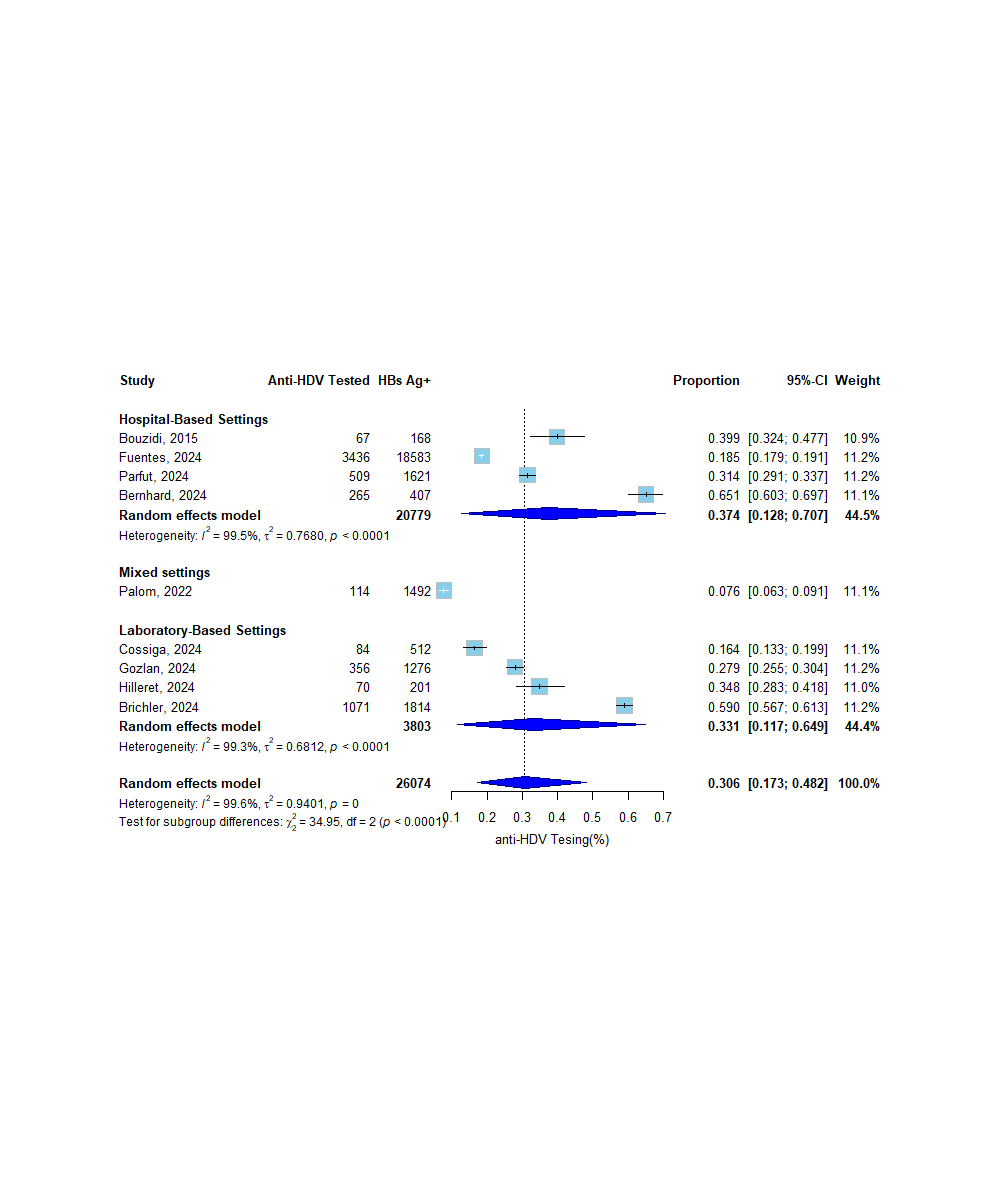
**

**b: Anti-HDV+ among who tested Anti-HDV**

**
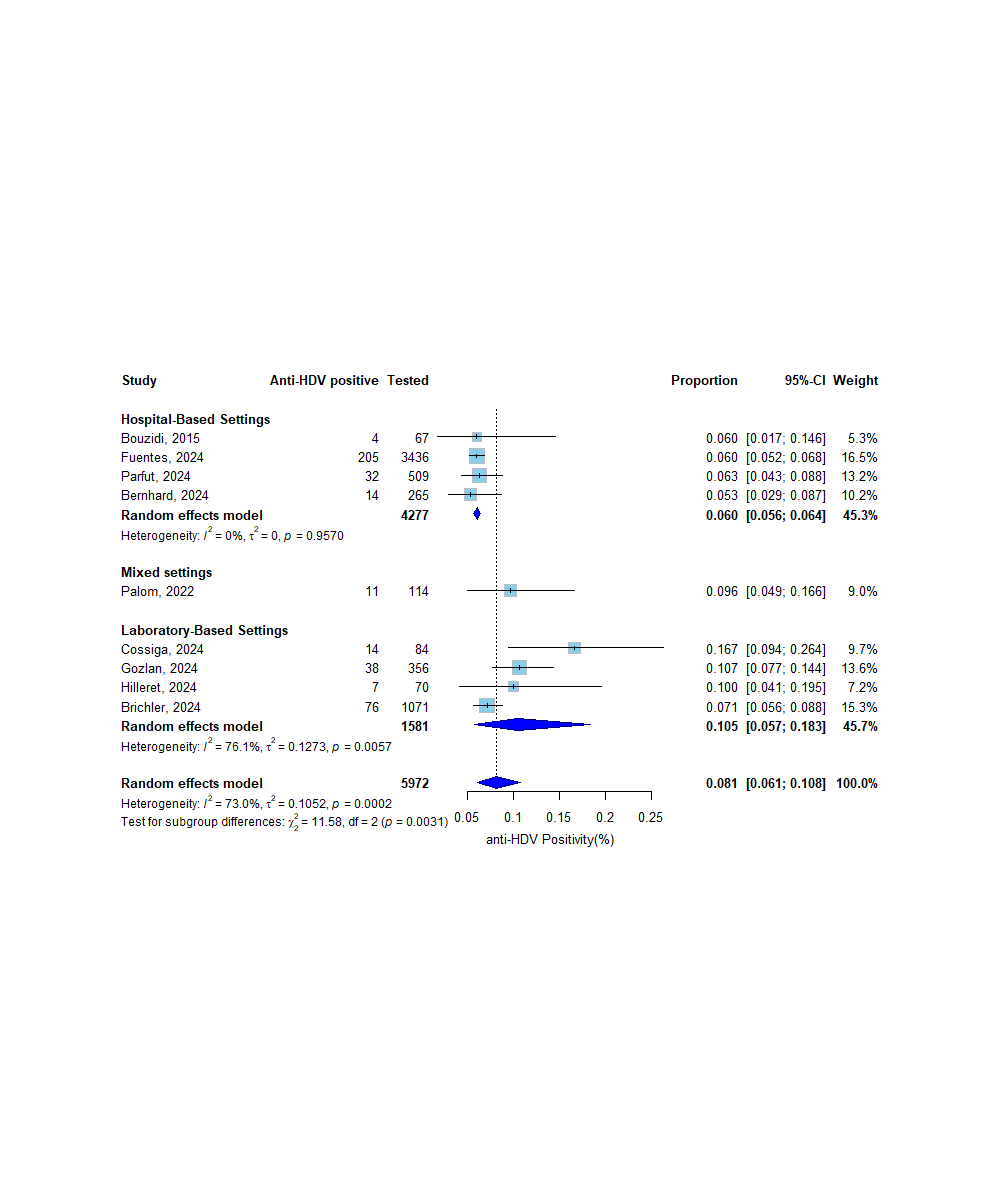
**

**c: HDV RNA tested among who Anti-HDV+**

**
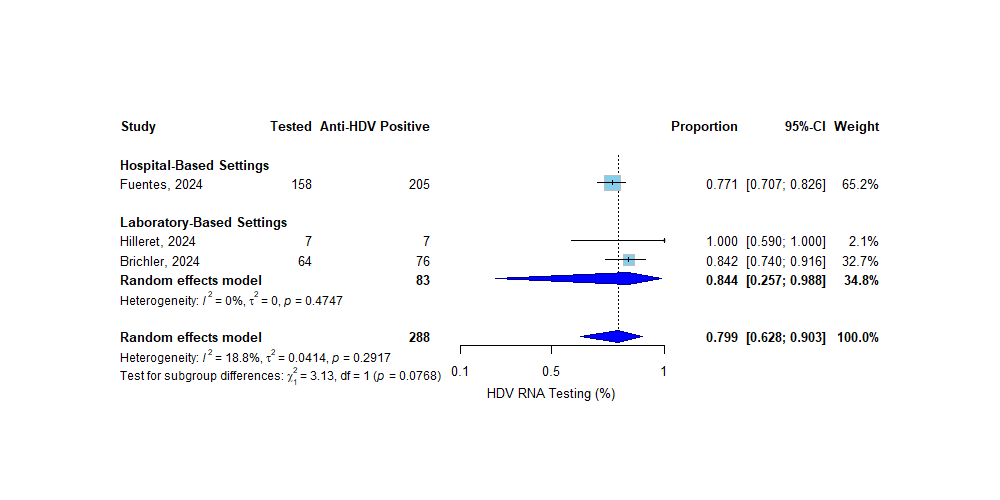
**

**Funnel plots:**

**
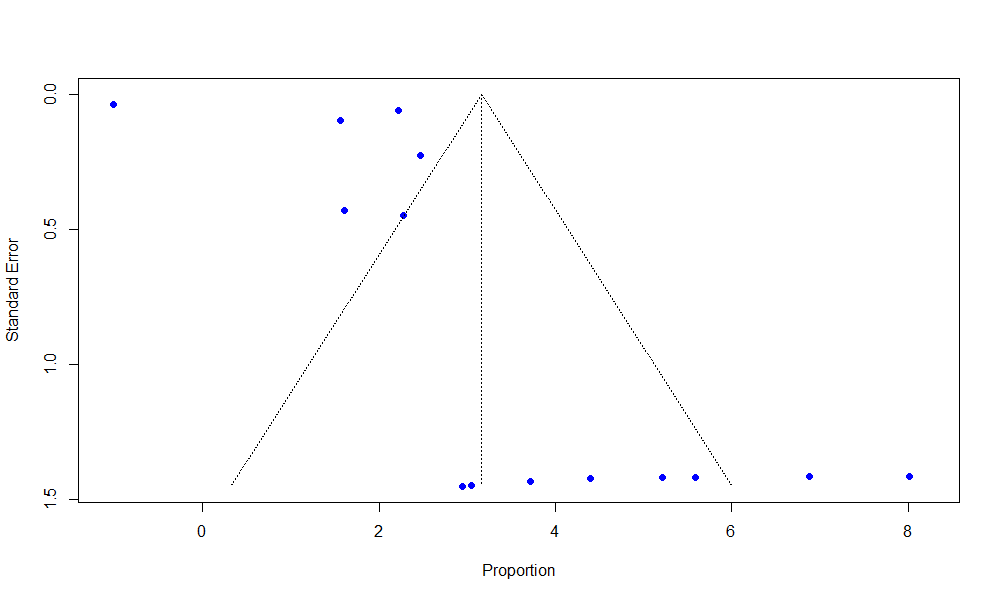
a. For HBs Ag+ among who tested for HBs Ag**

**b. For Anti-HDV tested among who HBs Ag+**

**
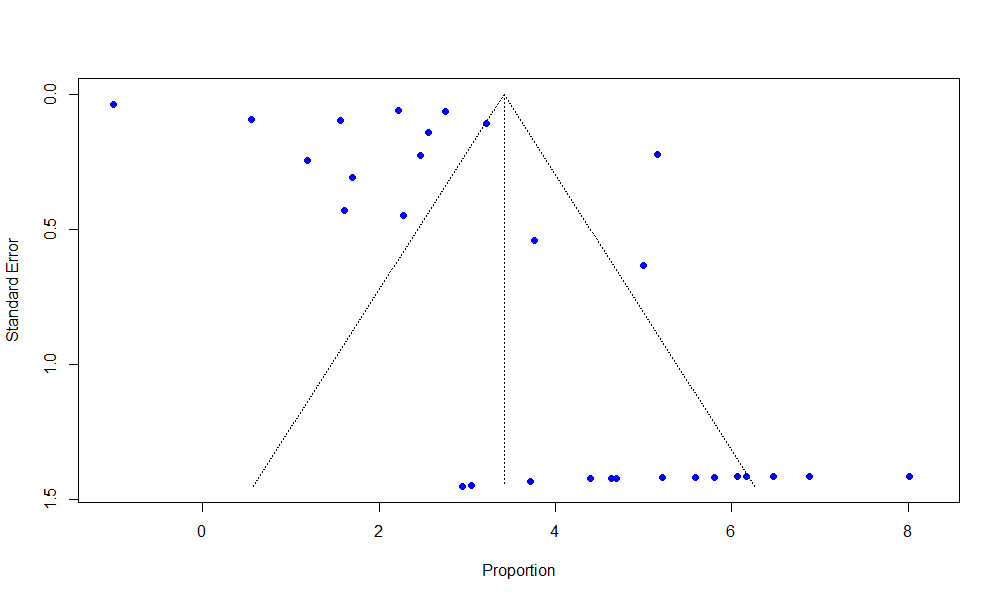
**

**c. For Anti-HDV+ among who tested Anti-HDV**

**
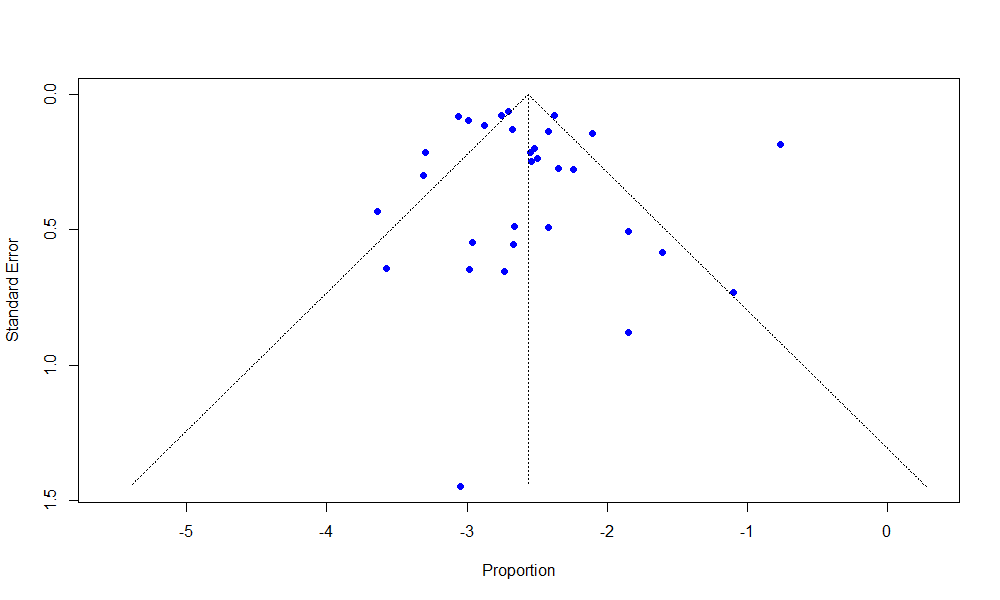
**

**d. For HDV RNA tested among who Anti-HDV+**

**
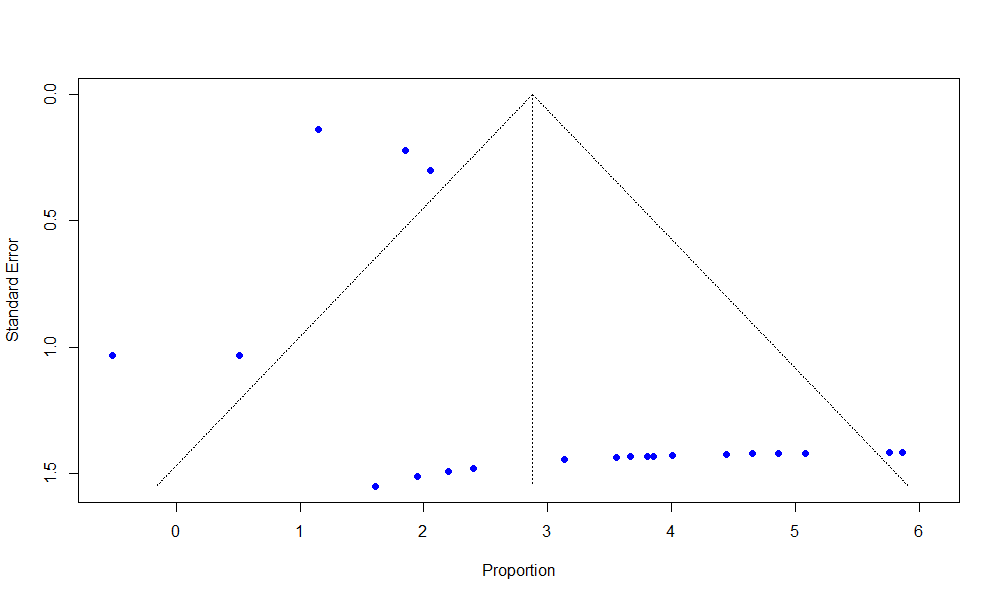
**

**e. For HDV RNA positive among who have HDV RNA test**

**
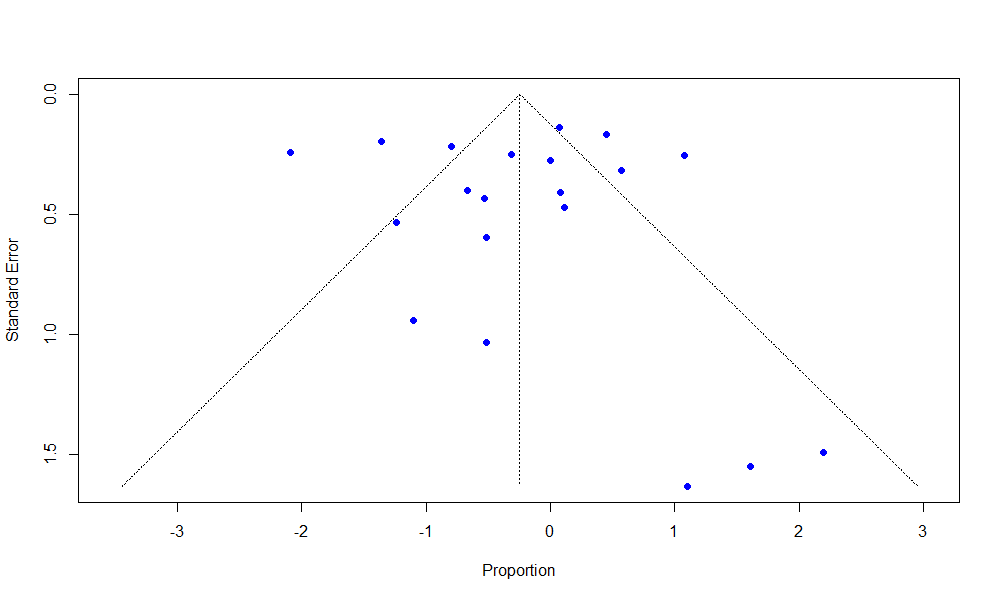
**

**f. For comparison of Anti-HDV testing between reflex and non-Reflex testing approaches**

**
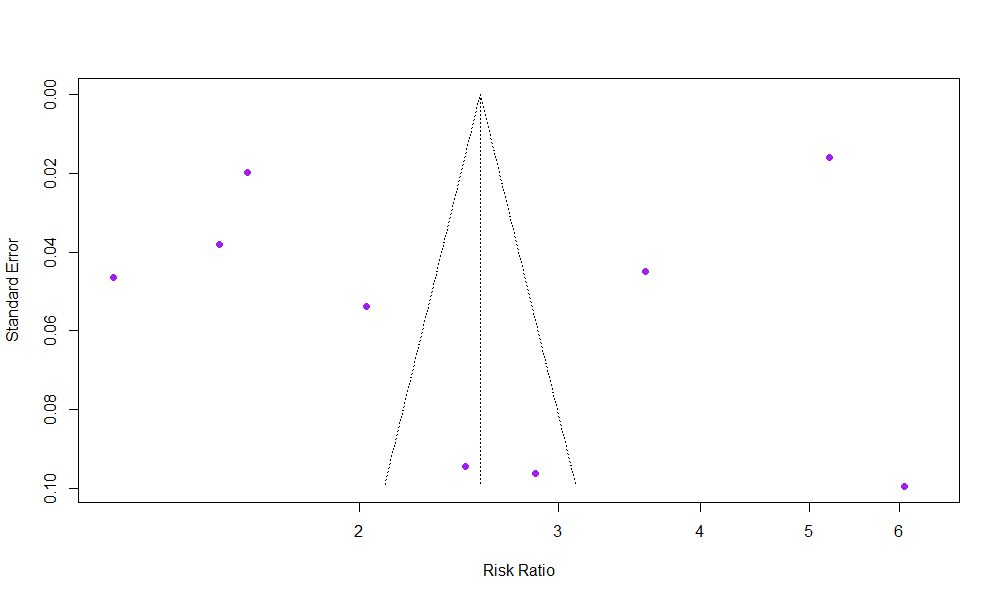
**

**g. For comparison of HDV RNA testing between reflex and non-Reflex testing approaches**

**
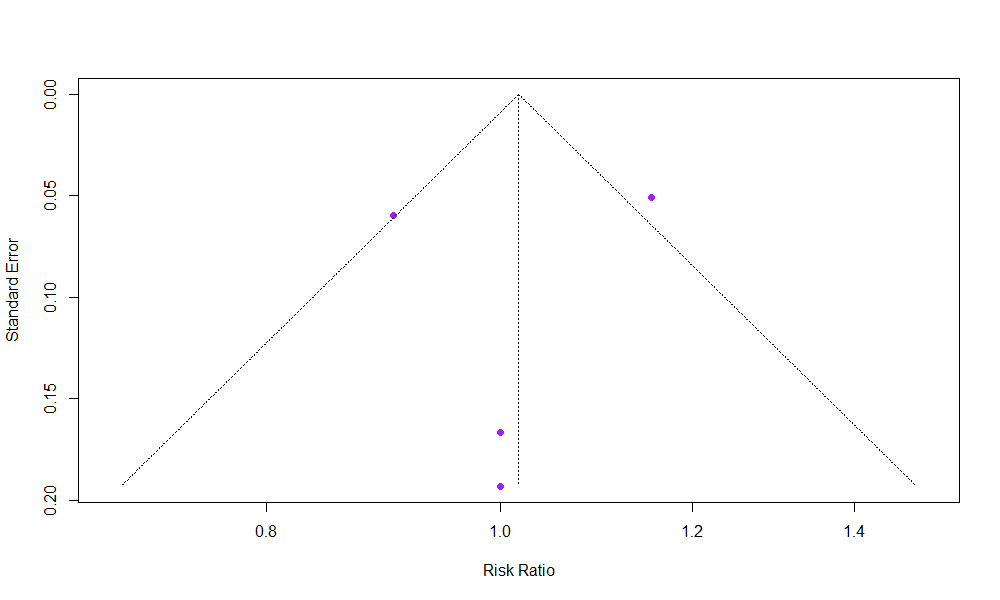
**

**h. For comparison of HDV RNA positivity rates between reflex and non-Reflex testing approaches**

**
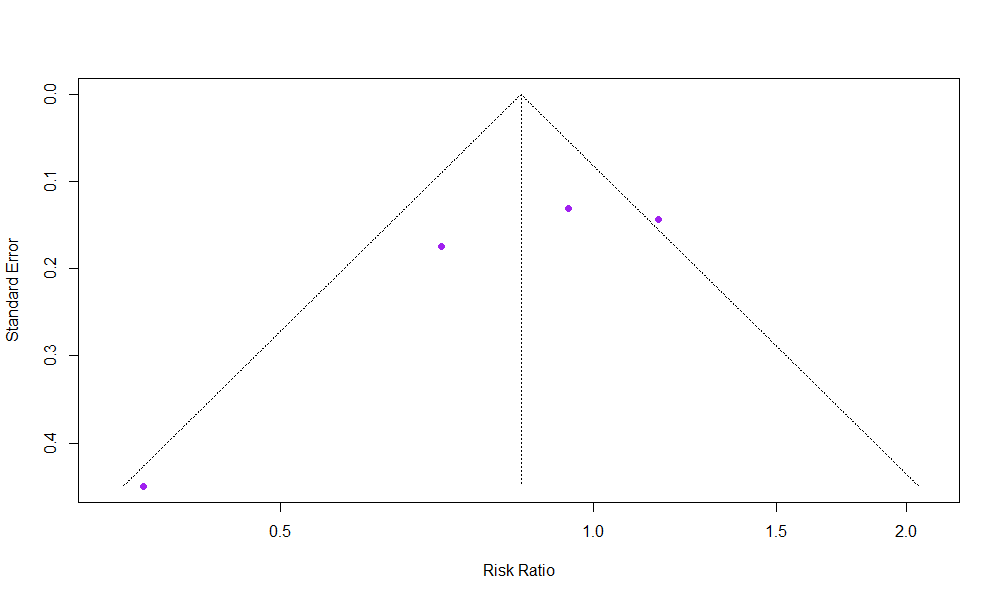
**
